# Spatial patterns of brain lesions assessed through covariance estimations of lesional voxels in multiple Sclerosis: The SPACE-MS technique

**DOI:** 10.1016/j.nicl.2021.102904

**Published:** 2021-12-02

**Authors:** Carmen Tur, Francesco Grussu, Floriana De Angelis, Ferran Prados, Baris Kanber, Alberto Calvi, Arman Eshaghi, Thalis Charalambous, Rosa Cortese, Declan T. Chard, Jeremy Chataway, Alan J. Thompson, Olga Ciccarelli, Claudia A.M. Gandini Wheeler-Kingshott

**Affiliations:** a NMR Research Unit, Queen Square MS Centre, Department of Neuroinflammation, UCL Queen Square Institute of Neurology, Faculty of Brain Sciences, University College London, UK; b MS Centre of Catalonia (Cemcat), Vall d’Hebron Institute of Research, Vall d’Hebron Barcelona Hospital Campus, Spain; c Radiomics Group, Vall d’Hebron Institute of Oncology, Vall d’Hebron Barcelona Hospital Campus, Barcelona, Spain; d Centre for Medical Image Computing, Medical Physics and Biomedical Engineering Department, University College London, UK; e e-Health Center, Universitat Oberta de Catalunya, Spain; f National Institute for Health Research University College London Hospitals Biomedical Research Centre, UK; g Department of Brain and Behavioural Sciences, University of Pavia, Italy; h Brain Connectivity Centre, IRCCS Mondino Foundation, Pavia, Italy

**Keywords:** Multiple sclerosis, Lesion spatial distribution, Magnetic resonance imaging, Anisotropy, Caudality, SPACE-MS

## Abstract

Predicting disability in progressive multiple sclerosis (MS) is extremely challenging. Although there is some evidence that the spatial distribution of white matter (WM) lesions may play a role in disability accumulation, the lack of well-established quantitative metrics that characterise these aspects of MS pathology makes it difficult to assess their relevance for clinical progression. This study introduces a novel approach, called *SPACE-MS,* to quantitatively characterise spatial distributional features of brain MS lesions, so that these can be assessed as predictors of disability accumulation. In SPACE-MS, the covariance matrix of the spatial positions of each patient’s lesional voxels is computed and its eigenvalues extracted. These are combined to derive rotationally-invariant metrics known to be common and robust descriptors of ellipsoid shape such as anisotropy, planarity and sphericity. Additionally, SPACE-MS metrics include a neuraxis caudality index, which we defined for the whole-brain lesion mask as well as for the most caudal brain lesion. These indicate how distant from the supplementary motor cortex (along the neuraxis) the whole-brain mask or the most caudal brain lesions are.

We applied SPACE-MS to data from 515 patients involved in three studies: the MS-SMART (NCT01910259) and MS-STAT1 (NCT00647348) secondary progressive MS trials, and an observational study of primary and secondary progressive MS. Patients were assessed on motor and cognitive disability scales and underwent structural brain MRI (1.5/3.0 T), at baseline and after 2 years. The MRI protocol included 3DT1-weighted (1x1x1mm^3^) and 2DT2-weighted (1x1x3mm^3^) anatomical imaging. WM lesions were semiautomatically segmented on the T2-weighted scans, deriving whole-brain lesion masks. After co-registering the masks to the T1 images, SPACE-MS metrics were calculated and analysed through a series of multiple linear regression models, which were built to assess the ability of spatial distributional metrics to explain concurrent and future disability after adjusting for confounders.

Patients whose WM lesions laid more caudally along the neuraxis or were more isotropically distributed in the brain (i.e. with whole-brain lesion masks displaying a high sphericity index) at baseline had greater motor and/or cognitive disability at baseline and over time, independently of brain lesion load and atrophy measures. In conclusion, here we introduced the SPACE-MS approach, which we showed is able to capture clinically relevant spatial distributional features of MS lesions independently of the sheer amount of lesions and brain tissue loss. Location of lesions in lower parts of the brain, where neurite density is particularly high, such as in the cerebellum and brainstem, and greater spatial spreading of lesions (i.e. more isotropic whole-brain lesion masks), possibly reflecting a higher number of WM tracts involved, are associated with clinical deterioration in progressive MS. The usefulness of the SPACE-MS approach, here demonstrated in MS, may be explored in other conditions also characterised by the presence of brain WM lesions.

## Highlights

• We present SPACE-MS, a tool to assess the spatial distribution of brain lesions.
• SPACE-MS metrics mainly reflect caudality and spatial spreading of brain lesions.
• More caudal and more widespread brain lesions correlate with worse disability.
• SPACE-MS metrics can be automatically obtained using routine anatomical scans.
• The usefulness of the SPACE-MS approach should be explored in other conditions.

## Graphical abstract

**Figure:**
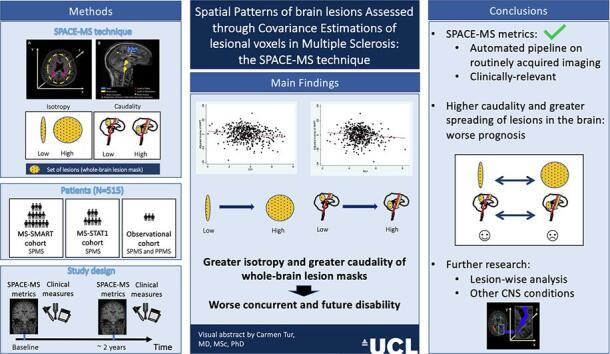

## 1 Introduction

In most neurological conditions characterised by the presence of white matter (WM) lesions in the central nervous system (CNS), such as multiple sclerosis (MS), CNS vasculitis, or small vessel disease (SVD), the extent of such lesions is strongly associated with the severity of the disease (Cannerfelt et al., 2018, Pantoni, 2010, Thompson et al., 2018, Tintore et al., 2015). However, in the clinic, they are assessed qualitatively, or at most, semi-quantitatively, limiting the assessment to a categorisation of the lesion number, e.g. low, medium and high lesion load (Tintore et al., 2015). Research studies have proposed metrics like lesion counts or volume, although their correlation with clinical outcomes is weaker than desired (Fisniku et al., 2008). This happens because, at least partly, they do not account for other, potentially crucial aspects of lesions such as their spatial location or the degree of tissue destruction caused by them (Grussu et al., 2017, Naismith et al., 2010, Absinta et al., 2019). Recently, the biological underpinning of lesions through quantitative MRI has started to be assessed (Absinta et al., 2019). However, a formal, quantitative characterisation of the spatial distribution of brain WM lesions has never been carried out, hampering the assessment of its relevance for clinical progression in neurological conditions. Inspired by geostatistics, here we propose a method developed to assess the spatial distribution of WM brain lesions and apply it to MS.

Our method, called *SPACE-MS,* is based on the estimation of the spatial variability (or spread) of lesional voxels in the brain. Information on the spatial variability is obtained from the covariance matrix constructed from the spatial position of lesional voxels along the x, y and z axis. Since such a covariance matrix is a rank-2 tensor, we used common descriptors of tensor shape, such as anisotropy, planarity and sphericity indices (Ennis and Kindlmann, 2006, Prados et al., 2010), based on the eigenvalues of the covariance matrix, to describe how brain lesional voxels, and therefore, lesions, spread.

The rationale behind the use of a tensor to characterise the spatial distribution of brain lesions stems from the elegance and robustness of the framework. The method has strong clinical appeal, as it relies on routine anatomical imaging that is performed for lesion assessment in virtually all radiology departments in the world. Here we demonstrate it in MS, but it could be used in any brain condition known to cause focal lesions in neural tissue.

MS is an inflammatory-demyelinating condition of the central nervous system that usually affects young adults and evolves over decades (Thompson et al., 2018). Its effects are difficult to predict in individuals, with some people accruing little disability and, at the other end of the scale, some having their life shortened by MS. WM brain lesion load, determined using magnetic resonance imaging (MRI), is currently one of the main predictors of future disability (Fisniku et al., 2008). However, whole-brain WM lesion loads explain a minority of disability accrual in MS, i.e. <50% in the best case scenario (Fisniku et al., 2008), and there are several potentially significant reasons for this. Ultimately it is neurodegeneration that is thought to explain the majority of irreversible disability in MS (Eshaghi et al., 2018), and while WM lesions can look very similar on conventional MRI scans, we know that histopathologically they are diverse, with some showing substantially more axonal damage than others, and some exhibiting ongoing inflammatory activity years after they first formed (Filippi et al., 2013, He et al., 2009, Eden et al., 2019, Dekker et al., 2020, Frischer et al., 2015). We also know that many WM lesions are clinically silent, and that the location of a lesion determines the likelihood that it will be clinically declared or clinically relevant (Eden et al., 2019). For instance, it is known that the presence of infratentorial (Tintore et al., 2010, Chung et al., 2020) or spinal cord lesions (Chung et al., 2020, Brownlee et al., 2019) increases the risk of disease progression after a first demyelinating attack (clinically isolated syndrome) or in established MS (Eden et al., 2019, Kerbrat et al., 2020). Brain connectivity research also points at a crucial role of the spatial distribution of brain lesions in the development of disability in MS, since the damage in specific WM fibre tracts may lead to a reduction in the efficiency of the brain network, independently of the lesion load (Charalambous et al., 2019). Along these lines, recent studies have also suggested that the spatial distribution of lesions may lead to specific patterns of disconnection between grey matter (GM) areas (Kanber et al., 2019) which can be particularly harmful.

In this work, we hypothesise that a greater spreading of lesions, and a more isotropic pattern of such spreading entail a worse prognosis, probably because they reflect a greater dissemination of the underlying demyelinating changes that occur in MS. Thus, our main aim was to assess whether covariance-based spatial distributional features of lesions were able to explain concurrent and future disability in progressive MS, and if they could do so independently of more common and widely accepted predictors, such as WM lesion load or brain atrophy measures (as markers of global neurodegeneration, and the main MRI outcome measures used in early phase progressive MS trials). Additionally, given that infratentorial and spinal cord lesions are known to carry a worse prognosis (Tintore et al., 2010, Chung et al., 2020, Brownlee et al., 2019), we assessed the impact of the location of the whole-brain lesion mask and of the most caudal lesion along the neural axis (or neuraxis) on the accumulation of disability.

## 2 Materials and methods

### 2.1 Theory: SPACE-MS metrics

Let us indicate with $C=cov\left(r\right)=E\left[rr^{T}\right]$ the covariance matrix of the 3D positions r of lesional voxels within a binary mask (either whole-brain mask or an individual lesion).

Let us also indicate with $u_{1}\geq u_{2}\geq u_{3}\geq 0$ the three eigenvalues of C, after performing a principal component analysis.

**Fig. 1:**
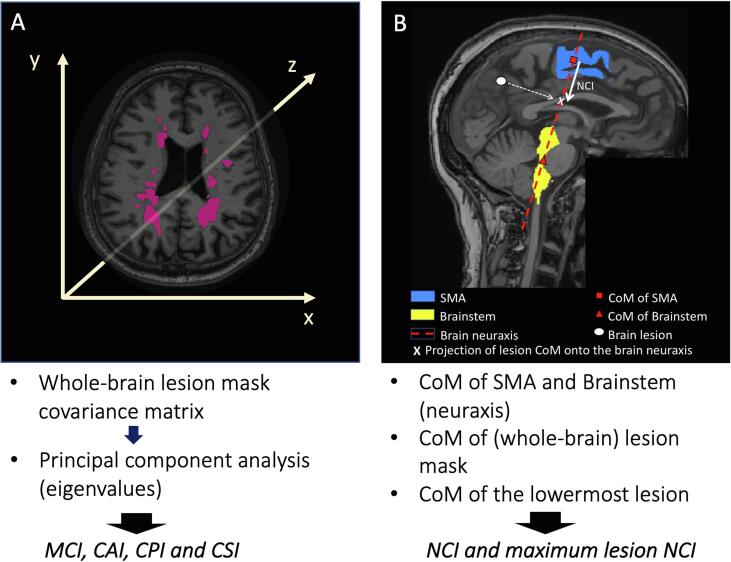
**Analysis pipeline A.** Once all the individual lesions were defined, the 3D positions of all lesional voxels within the whole-brain mask were extracted. These 3D positions were used to evaluate their spatial covariance matrix, from which the eigenvalues were computed after performing a principal component analysis. The eigenvalues were then used to obtain some of the spatial distribution metrics (see Methods section). **B.** This figure shows the definition of neuraxis and of neuraxis caudality index (NCI) for a given lesion (lesion NCI). The whole-brain NCI was computed in the same way, substituting the CoM of each individual lesion for the CoM of the whole-brain lesion mask. The maximum lesion NCI was computed calculating the CoM of the lowermost brain lesion. *Abbreviations (in alphabetical order)*: CAI: Covariance Anisotropy Index; CoM: centre of mass; CPI: Covariance Planarity Index; CSI: Covariance Sphericity Index; MCI: Mean Covariance Index; NCI: neuraxis caudality index; SMA: supplementary motor area.

**Fig. 2:**
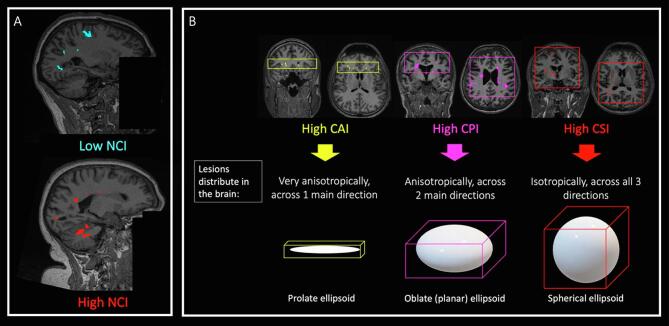
**Examples of spatial distribution metrics**. This figure shows the significance of our spatial distribution metrics. In A, there is an example of a lesion mask with low NCI (whose CoM lies very close to the SMA) and another example of a lesion mask with very high NCI (whose CoM lies very close to the brainstem). In B, there are schematic descriptions of different types of lesion masks, with some real examples: on the left, a whole-brain lesion mask with a very high CAI, where lesions would spread anisotropically, mainly across one spatial direction; in the middle, a lesion mask with a very high CPI, where lesions would spread mainly across two directions; on the right, a lesion mask with a very high CSI, where lesions would spread isotropically, i.e. across all three spatial directions almost equally. *Abbreviations*: CAI: covariance anisotropy index; CPI: covariance planarity index; CSI: covariance sphericity index; NCI: neuraxis caudality index.

SPACE-MS metrics are rotationally-invariant indices derived by combining the eigenvalues of C, which aim to capture the spatial variation of the lesional voxel positions. Moreover, in SPACE-MS the distribution of the damage along the neuraxis is also evaluated, since this is thought to be clinically relevant (Kerbrat et al., 2020). To this end, SPACE-MS relies on the coordinates of the centre of mass (CoM), i.e. the point representing the centre (mean position) of any 3D object, of the 1) (whole-brain) lesion mask; 2) lowermost brain lesion; 3) unified supplementary motor areas (SMAs), i.e. right and left considered as one; and 4) brainstem (Fig. 1 and Fig. 2).

## 3 Computation of SPACE-MS metrics

1) Position along the neuraxis:

This is evaluated through the Neuraxis Caudality Index (NCI), a novel metric which, to the best of our knowledge, is described here for the first time. This is the *normalised* distance between the CoM of both SMAs and the projection of the lesion mask CoM onto the neuraxis. In SPACE-MS, the neuraxis is defined as the line joining the CoM of both SMAs and the CoM of the brainstem (Fig. 1). The *normalisation* of this distance was performed through dividing it by the distance between the CoM of both SMAs and the CoM of the brainstem, to avoid the potential confounding effect of head size:

(1) $$NCI=\frac{{\left(r_{mask}-r_{SMA}\right)}^{T}\left(r_{BS}-r_{SMA}\right)}{{\|r_{BS}-r_{SMA}\|}^{2}},$$

In (1), $r_{mask}=E\left[r\right]$ is the CoM of the mask, $r_{SMA}$ is the CoM of the supplementary motor area, and $r_{BS}$ is the CoM of the brainstem. The transposition operator ${∙}^{T}$ enables computation of the dot product. NCI describes the normalised (by head size) distance between $r_{mask}$ measured from $r_{SMA}$ along the spatial direction $n=\frac{\left(r_{BS}-r_{SMA}\right)}{\|r_{BS}-r_{SMA}\|}$ identifying the neuraxis. NCI = 0 implies that the projection of $r_{mask}$ onto n is exactly at the level of the CoM of the supplementary motor area, while NCI = 1 implies that the projection of $r_{mask}$ onto n is exactly at the level of the CoM of the brainstem. In theory, NCI could range in [-∞; +∞], although in practice it is expected to range approximately between 0 and 1.5, given the anatomical constraints. Thus, higher values of NCI indicate a higher distance of the whole-brain lesion mask from the SMA (Fig. 2).

NCI can be calculated for the entire brain lesion mask, or for a specific lesion. Given the known clinical relevance of having a very caudal lesion in the brain, namely in the brainstem or the cerebellum, independently of where the rest of the lesions lie (Tintore et al., 2010, Chung et al., 2020), in this work we also explore the utility of NCI of the *lowermost brain lesion.* We called this distance *maximum* lesion NCI.

2) Total amount of spatial variability:

This is measured by the *Mean Covariance Index (MCI)*, which is computed as the mean of the three eigenvalues of the covariance matrix, *u_1_*, *u_2_* and *u_3_*:

(2) $$MCI=\frac{u_{1}+u_{2}+u_{3}}{3},$$

In (2), MCI ranges in [0; +∞], with increasing MCI implying higher spatial variability. The theoretical framework of this metric is not new, and has been applied before to diffusion-weighted imaging data (Ennis and Kindlmann, 2006). However, its use to characterise (whole-brain) lesion masks is completely new.

3) number of spatial dimensions across which spatial variability spreads:

For this purpose, we use well-known descriptors of ellipsoid shape, which we called CAI, CPI and CSI, and which are explained below (Ennis and Kindlmann, 2006, Prados et al., 2010). Yet its use for the characterisation of whole-brain lesion masks is new.

a) One dimension: this is evaluated through the *Covariance Anisotropy Index (CAI),* a scalar value between 0 and 1 describing how anisotropic the lesion mask is, conceptually affine to well-known diffusion tensor MRI fractional anisotropy (Basser et al., 1994). Values close to 1 indicate that there is a dominant direction across which the lesional voxels spread (i.e. lesional voxels spread across a prolate ellipsoid) (Fig. 2).

(3) $$CAI=\frac{\sqrt{3}}{\sqrt{2}}\frac{\sqrt{{\left(u_{1}-u\right)}^{2}+{\left(u_{2}-u\right)}^{2}+{\left(u_{3}-u\right)}^{2}}}{\sqrt{u_{1}^{2}+u_{2}^{2}+u_{3}^{2}}},$$

In (3), $u=\frac{\left(u_{1}+u_{2}+u_{3}\right)}{3}$. CAI ranges in [0; 1], with increasing CAI implying higher anisotropy.

b) Two dimensions: this is evaluated through the *Covariance Planarity Index (CPI)*, a scalar value between 0 and 1 describing how similar to a plane the ellipsoid defined by the limits of the lesion mask is. Values close to 1 indicate that the lesion mask is mainly distributed on a plane, with the variability spreading across two main spatial dimensions (i.e. lesional voxels spread across an oblate ellipsoid) (Fig. 2).

(4) $$CPI=\frac{2\left(u_{2}-u_{3}\right)}{\left(u_{1}+u_{2}+u_{3}\right)},$$

In (4), CPI ranges in [0;1], with increasing CPI implying higher planarity.

c) Three dimensions: this is evaluated through the *Covariance Sphericity Index (CSI),* a scalar value between 0 and 1 describing to what extent the lesional voxels of the lesion mask spread across all three spatial dimensions or not. CSI values close to 1 indicate that the lesional voxels distribute across the three spatial directions very similarly (i.e. lesional voxels spread across a spherical ellipsoid) (Fig. 2).

(5) $$CSI=\frac{3u_{3}}{\left(u_{1}+u_{2}+u_{3}\right)},$$

In (5), CSI ranges in [0; 1], with increasing CSI implying higher spherical shape/distribution of the lesional voxels (i.e. higher likelihood of all 3 spatial directions being involved in lesion spreading).

### 3.1 Subjects

We included patients with progressive MS from three independent longitudinal cohorts: the MS-SMART (NCT01910259) (N = 356) (Chataway et al., 2020) and the MS-STAT (NCT00647348) (N = 126) (Chataway et al., 2014) trial cohorts, and an observational cohort (N = 33) (Charalambous et al., 2019). Before starting any of these studies, patients gave written consent for their data to be used in subsequent studies, which were approved by the local Ethics Committee.

The MS-SMART trial was a multicentre, multiarm, randomised, double-blind, placebo-controlled, parallel-group phase IIb trial, where 445 patients with secondary progressive MS from 13 UK clinical neuroscience centres were randomised to receive amiloride, fluoxetine, riluzole or placebo as putative neuroprotective drugs. (Chataway et al., 2020) No differences were observed between treatment arms in the primary endpoint (whole-brain volume change), as has been published elsewhere (Chataway et al., 2020, De Angelis et al., 2020). The participants included in this trial underwent 1.5/3T MRI scans at baseline and at 24-week and 96-week follow-up. They were also assessed clinically at baseline and yearly thereafter on the Expanded Disability Status Scale (EDSS) (Kurtzke, 1983), the Timed 25-foot Walk Test (TWT) (Cutter, 1999), the Nine Hole Peg Test (9HPT) (Cutter, 1999), the Paced Auditory Serial Addition Test (PASAT) (Cutter, 1999) and the Symbol Digit Modalities Test (SDMT) (Smith, 2007). For this study, out of the initial 445 participants, we included those 356 who had attended MRI sessions and clinical assessments at all time points. For the statistical analyses described below, the *clinical variables* (and their units) used were: *EDSS score* (EDSS score points), *inverse of the TWT,* i.e. reciprocal of the mean of the two attempts (1/s), the *inverse of the 9HPT,* i.e. average of the reciprocal value of the mean time of the two right-hand attempts and the reciprocal value of the mean time of the two left-hand attempts (1/s), the *PASAT score* (number of correct answers) and the *SDMT score* (number of correct answers).

The MS-STAT trial was a multicentre, randomised, double-blind, placebo-controlled, parallel-group phase II trial, where 140 patients with secondary progressive MS from three neuroscience centres (but only two scanning centres) were randomised to receive simvastatin or placebo (Chataway et al., 2014). Patients underwent clinical and MRI assessments at baseline and at years 1 and 2. Out of the initial 140 participants, we included those 126 who attended baseline and 2-year MRI sessions and clinical assessments. Clinical outcomes included: EDSS, TWT, 9HPT and PASAT scores, which were treated as described for the MS-SMART cohort.

The observational cohort consisted of 33 patients with progressive MS (14 primary progressive MS and 19 secondary progressive MS) (Charalambous et al., 2019, Sethi et al., 2016). This is a progressive MS sub-cohort of a larger cohort, whose details have been presented in the past (Charalambous et al., 2019, Sethi et al., 2016). Patients underwent clinical and MRI assessments at baseline and after 20 months (standard deviation: 5.8) of follow-up (Sethi et al., 2016). Clinical variables included the EDSS and SDMT scores at baseline (N = 33). At follow-up, all patients were scored on the EDSS, but only 15 patients were scored on the SDMT.

### 3.2 MRI acquisition

MRI acquisition protocols of the MS-SMART trial included: i) 3D T1-weighted images (voxel resolution: 1 × 1 × 1 mm^3^), ii) axial dual echo fast/turbo spin echo proton density (PD)/T2-weighted from foramen magnum to vertex (1 × 1 × 3 mm^3^), as well as other sequences, not used in this study (Connick et al., 2018). MRI scanners included Philips 1.5 T/3T, Siemens 1.5 T/3T and GE 1.5 T/3T. The full MRI protocol of the MS-SMART trial has been published elsewhere (Connick et al., 2018).

MRI acquisition protocols of the MS-STAT trial have been reported elsewhere (Chataway et al., 2014). Two scanners (General Electric (GE) 3 Tesla and Siemens 1.5 Tesla) were used in the study, but each patient was scanned consistently with the same machine throughout the trial. The protocol included 3D (volumetric) T1-weighted images (GE scanner: 0.976 × 0.976 × 1.1 mm^3^; Siemens scanner: 1.25 × 1.25 × 1.2 mm^3^), and dual-echo fast/turbo spin echo T2-weighted images (1 × 1 × 3 mm^3^).

The MRI protocol for the observational cohort included 3D T1-weighted images with a fast-field echo scan (1 × 1 × 1 mm^3^) and dual-echo proton density/T2-weighted axial oblique images (1 × 1 × 3 mm^3^), in a 3 T Philips Achieva MR scanner.

### 3.3 MRI pre-processing

All the data underwent the same pre-processing pipeline. Pre-processing steps included: (1) semi-automated segmentation of WM lesions in T2-weighted images: T2 hyperintense lesions were identified manually but using a semi-automated edge finding tool (JIM v7.0, Xinapse Systems, Aldwincle, UK); (2) rigid co-registration of T2-weighted follow-up scans to T2-weighted scan at baseline in order to propagate the lesions to the follow-up scans; (3) resampling of lesion mask images to isotropic 3D T1 space; (4) lesion filling of 3D T1-weighted image, (Prados et al., 2016) and (5) subsequent Geodesic Information Flows (GIF) brain region segmentation; (Cardoso et al., 2015) (6) computation, for each subject and time point, of whole-brain lesion load. Additionally, normal-appearing (i.e. lesion-free) WM and GM volumes, including deep and cortical GM volumes, were also obtained, through GIF segmentation; (Cardoso et al., 2015) (7) extraction of bilateral (merged) SMA and brainstem masks, needed to compute NCI and maximum lesion NCI metrics: based on GIF brain parcellations, both SMAs were identified and a unified mask containing only this tissue, bilaterally, was created; the brainstem was also identified on the GIF parcellation, and a mask containing this tissue was also created. These two masks were used to estimate the neuraxis (as explained above).

### 3.4 Quantitative characterisation of spatial distribution of MS lesions

**Fig. 3:**
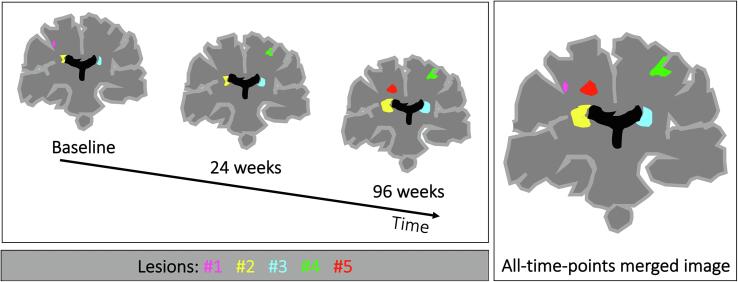
**Lesion detection across time points.** This figure shows how individual lesions were defined on the whole-brain lesion mask. Independent connected components were detected in a lesion mask obtained by merging the lesions masks corresponding from different time points (referred to as *all-time-points merged image*).

Individual lesions were defined by labelling independent connected components on the whole-brain lesion mask in an *all-time-points merged image* (Fig. 3). This *all-time-points merged image* was generated in view of possible longitudinal analyses, since it allowed us to assign a consistent identifier to lesions from subsequent timepoints. Connected components were defined based on 3-connectivity, so that the maximum number of orthogonal hops required to consider a pixel/voxel as a neighbour in 3D space was equal to 3. Whenever during the follow-up two (or more) lesions merged into a larger one, we considered the original individual lesions as if they were only one, at all the time points. Then, at each time point, we extracted the 3D positions $r={\left[\begin{array}{ccc} x & y & z \end{array}\right]}^{T}$ of all lesional voxels within the whole-brain *lesion mask*. Afterwards we used these 3D positions to evaluate their spatial covariance matrix, and calculate SPACE-MS metrics as described in the previous section. These metrics were computed on native (3DT1) space in order to avoid any non-linear registration of the images that could alter the spatial distribution of lesions. However, in a subset of patients, i.e., all patients from the ‘Observational cohort’ (N = 32), we also computed them on a common, unbiased space, the Montreal Institute of Neurology (MNI) space. For that, we first obtained the lesion-filled T1 scans, which were co-registered to the MNI brain. We then applied the transformation matrix to the lesion masks, from which we obtained the SPACE-MS metrics. This was done as a sensitivity analysis which aimed at exploring the robustness of these metrics.

Of note, SPACE-MS metrics may also be computed on individual lesions. Although this was not the main focus of this paper, we also computed lesion-wise SPACE-MS metrics, in addition to whole-brain ones, as an exploratory analysis (see Supplementary Fig. 1).

### 3.5 Statistical analyses

#### 3.5.1 Descriptive statistics and preliminary analyses

For all SPACE-MS metrics, common descriptors of variable distributions were obtained. Pearson’s (or Spearman’s, when appropriate) correlation coefficients were computed to assess associations between SPACE-MS metrics and demographic, clinical and conventional MRI variables at baseline, adjusting for scanning centre.

#### 3.5.2 SPACE-MS metrics to explain disability measures at baseline

To understand the added value of spatial distribution metrics to conventional predictors of disability, we built a series of multiple linear regression models with baseline disability metrics at baseline as the dependent variable (one at a time), as follows:

1) ‘Best *a priori* model’ of concurrent disability (with available data), including confounders but not including SPACE-MS metrics:

(6) $$y_{i}=\beta_{0}+\beta_{1}{\left[lesionvolumeatBL\right]}_{i}+\beta_{2}{\left[GMvolumeatBL\right]}_{i}+\beta_{3}{\left[WMvolumeatBL\right]}_{i}+\beta_{4}{\left[ageatBL\right]}_{i}+\beta_{5}{\left[malesex\right]}_{i}+\beta_{6}{\left[diseasedurationatBL\right]}_{i}+\sum_{k=1}^{16}\beta_{7.k}{\left[{\mathrm{centre}}_{k}\right]}_{i}+\varepsilon_{i},where\varepsilon_{i}∼NID\left(0,\sigma^{2}\right)$$

$y_{i}=$ clinical variable at baseline for ${\mathrm{subject}}_{i}$, i.e. either EDSS, inverse of TWT, inverse of NHPT, PASAT raw score, or SDMT raw score for that ${\mathrm{subject}}_{i}$; BL = baseline; $\beta_{0}=$ intercept; $\sum_{l=1}^{22}\beta_{l}=$ regression coefficients of the model (6 + 16 = 22). For ‘study centre’, 16 different dummy variables were created, one for each study centre whose data have been analysed; thus, the model produced 16 centre-specific regression coefficients (not reported); $\varepsilon_{i}=$ random error (or true residual) for the *i^th^* observation, which represents the difference between the observed value and its predicted value according to the model; true residuals ($\varepsilon_{i}$) are assumed to be normally and independently distributed. All this applies to all models below.

These models were built based on previous publications showing the importance of high brain WM lesion loads, and low GM and WM brain volumes for disability accrual in MS (Fisniku et al., 2008, Eshaghi et al., 2018, Brownlee et al., 2019, Haider et al., 2021, Tur et al., 2011, Conti et al., 2021). Additionally, these models took into account (older) age, (male) sex, and (longer) disease duration, which have been associated with (greater) risk of disability accrual (Tintore et al., 2015).

Therefore, we built five models, one for each clinical variable. For each of these, the R^2^ of the model, which indicates the percentage of the variability of the dependent variable that is explained by the model, was recorded.

2) ‘SPACE-MS models’ of concurrent disability (with available data), adding one SPACE-MS metric at a time:

(8) $$y_{i}=\beta_{0}+\beta_{1}{\left[lesionvolumeatBL\right]}_{i}+\beta_{2}{\left[GMvolumeatBL\right]}_{i}+\beta_{3}{\left[WMvolumeatBL\right]}_{i}+\beta_{4}{\left[ageatBL\right]}_{i}+\beta_{5}{\left[malesex\right]}_{i}+\beta_{6}{\left[diseasedurationatBL\right]}_{i}+\sum_{k=1}^{16}\beta_{7.k}{\left[{\mathrm{centre}}_{k}\right]}_{i}+\beta_{8}{\left[SPACEMSmetricatBL\right]}_{i}+\varepsilon_{i},where\varepsilon_{i}∼NID\left(0,\sigma^{2}\right)$$

Notation: please see explanations below equation (6). Since there are 6 SPACE-MS metrics, we built 5×6models, i.e. one for each clinical variable and for each SPACE-MS metric. For each of these models, the R^2^ of the model was also recorded.

Afterwards, the % increase in model performance (% improvement [%I]) was computed as:

(7) $$\%I=\frac{{(R}^{2}ofmodelwithSPACEMS-R^{2}\mathrm{ofmodelwithoutSPACEMS})}{R^{2}ofmodelwithoutSPACEMS}\times 100$$

As a second step, a sensitivity analysis was run by building such ‘Best *a priori* models’ through a more classical backward elimination strategy, where all potential predictors (except the SPACE-MS metric) were initially introduced together in the model. These were subsequently eliminated, one by one, based on their p-value, so that variables with non-significant p-values were eliminated first, until only significant (p < 0.05) were included as predictors. Afterwards, the SPACE-MS metrics (one at a time) were introduced in the model and the %I was computed as explained in equation (7).

3) ‘Best SPACE-MS model’ of concurrent disability (with available data), selecting the best set of SPACE-MS metrics:

(9) $$y_{i}=\beta_{0}+\beta_{1}{\left[lesionvolumeatBL\right]}_{i}+\beta_{2}{\left[GMvolumeatBL\right]}_{i}+\beta_{3}{\left[WMvolumeatBL\right]}_{i}+\beta_{4}{\left[ageatBL\right]}_{i}+\beta_{5}{\left[malesex\right]}_{i}+\beta_{6}{\left[diseasedurationatBL\right]}_{i}+\sum_{k=1}^{16}\beta_{7.k}{\left[{\mathrm{centre}}_{k}\right]}_{i}+\sum_{m=1}^{6}\beta_{8.m}{\left[SPACEMSmetricatBL\right]}_{i}+\varepsilon_{i},where\varepsilon_{i}∼NID\left(0,\sigma^{2}\right)$$

Notation: please see explanations below equation (6). For each dependent variable, we built a model similar to that described in equation (8), but including all SPACE-MS metrics at once. Then, stepwise backward selection of the SPACE-MS metrics was done, eliminating first those with highest p-values, and retaining in the model only those SPACE-MS metrics with p-values ≤ 0.05. After this step, one ‘final best SPACE-MS model’ for each clinical variable was obtained. Whenever the R^2^ of the model increased after the inclusion of the (best set of) SPACE-MS metrics, we considered that there was a model improvement.

#### 3.5.3 SPACE-MS metrics to predict future disability

Similar models as above were built, but including the clinical measure at the last time point as the dependent variable (one at a time). These models also included the clinical variable at baseline (BL) as a predictor. Thus, the models were:

1) ‘Best *a priori* model’ of future disability, not including SPACE-MS metrics:

(10) $$y_{i}=\beta_{0}+\beta_{1}{\left[lesionvolumeatBL\right]}_{i}+\beta_{2}{\left[GMvolumeatBL\right]}_{i}+\beta_{3}{\left[WMvolumeatBL\right]}_{i}+\beta_{4}{\left[ageatBL\right]}_{i}+\beta_{5}{\left[malesex\right]}_{i}+\beta_{6}{\left[diseasedurationatBL\right]}_{i}+\sum_{k=1}^{16}\beta_{7.k}{\left[{\mathrm{centre}}_{k}\right]}_{i}+\beta_{8}{\left[clinicalvariableatBL\right]}_{i}+\varepsilon_{i},where\varepsilon_{i}∼NID\left(0,\sigma^{2}\right)$$

$y_{i}=$ clinical variable at follow-up for ${\mathrm{subject}}_{i}$, i.e. either EDSS, inverse of TWT, inverse of NHPT, PASAT raw score, or SDMT raw score for that ${\mathrm{subject}}_{i}$; BL = baseline; for the rest of the notation, please see explanations below equation (6). As above, we built 5 models, one for each clinical variable.

2) ‘SPACE-MS models’ of future disability, adding one SPACE-MS metric at a time:

(11) $$y_{i}=\beta_{0}+\beta_{1}{\left[lesionvolumeatBL\right]}_{i}+\beta_{2}{\left[GMvolumeatBL\right]}_{i}+\beta_{3}{\left[WMvolumeatBL\right]}_{i}+\beta_{4}{\left[ageatBL\right]}_{i}+\beta_{5}{\left[malesex\right]}_{i}+\beta_{6}{\left[diseasedurationatBL\right]}_{i}+\sum_{k=1}^{16}\beta_{7.k}{\left[{\mathrm{centre}}_{k}\right]}_{i}+\beta_{8}{\left[SPACEMSmetricatBL\right]}_{i}+\beta_{9}{\left[clinicalvariableatBL\right]}_{i}+\varepsilon_{i},where\varepsilon_{i}∼NID\left(0,\sigma^{2}\right)$$

$y_{i}=$ clinical variable at follow-up for ${\mathrm{subject}}_{i}$, i.e. either EDSS, inverse of TWT, inverse of NHPT, PASAT raw score, or SDMT raw score for that ${\mathrm{subject}}_{i}$; BL = baseline; for the rest of the notation, please see explanations below equation (6). Here we built 5×6models, as above, one for each clinical variable and for each SPACE-MS metric.

Here, we also computed %I as shown in equation (7). Additionally, we built similar models but using a backward elimination strategy as described above.

3) ‘Best SPACE-MS model’ of future disability (with available data), selecting the best set of SPACE-MS metrics:

(12) $$y_{i}=\beta_{0}+\beta_{1}{\left[lesionvolumeatBL\right]}_{i}+\beta_{2}{\left[GMvolumeatBL\right]}_{i}+\beta_{3}{\left[WMvolumeatBL\right]}_{i}+\beta_{4}{\left[ageatBL\right]}_{i}+\beta_{5}{\left[malesex\right]}_{i}+\beta_{6}{\left[diseasedurationatBL\right]}_{i}+\sum_{k=1}^{16}\beta_{7.k}{\left[{\mathrm{centre}}_{k}\right]}_{i}+\sum_{m=1}^{6}\beta_{8.m}{\left[SPACEMSmetricatBL\right]}_{i}+\beta_{9}{\left[clinicalvariableatBL\right]}_{i}+\varepsilon_{i},where\varepsilon_{i}∼NID\left(0,\sigma^{2}\right)$$

$y_{i}=$ clinical variable at follow-up for ${\mathrm{subject}}_{i}$, i.e. either EDSS, inverse of TWT, inverse of NHPT, PASAT raw score, or SDMT raw score for that ${\mathrm{subject}}_{i}$; BL = baseline; for the rest of the notation, please see explanations below equation (6). To find the ‘best SPACE-MS model’ of future disability, we followed the same steps as those explained below equation (9) (stepwise backward selection of the SPACE-MS metrics).

#### 3.5.4 Dynamic changes in spatial distribution metrics

Patients belonging to the two trial cohorts had three MRI evaluations over time: at baseline, 6 months, and 2 years of follow-up (for the ‘MS-SMART’ cohort); and at baseline, 1 year and 2 years (for the ‘MS-STAT’ cohort). Instead, patients from the observational cohort only had two assessments, at baseline and after 20 months. For this, mixed-effects (longitudinal) models accounting for repeated measures, with random intercept and random slope for time (in years) were built. In these models, the spatial distribution metric computed at each time point was considered as the dependent variable (one at a time), and ‘time’ in years was the main explanatory variable. Additionally, these models included, as covariates, changes in lesion load between baseline and follow-up, lesion load and GM and WM volumes at baseline, age, sex, and disease duration at baseline.

13) $$y_{\mathrm{ij}}={(\beta }_{0}+u_{0j})+{(\beta }_{1}+u_{1j}){\left[timeinyears\right]}_{\mathrm{ij}}+\beta_{2}{\left[changesinlesionvolumebetweenBLandFU\right]}_{j}+\beta_{3}{\left[lesionvolumeatBL\right]}_{j}+\beta_{4}{\left[GMvolumeatBL\right]}_{j}+\beta_{5}{\left[WMvolumeatBL\right]}_{j}+\beta_{6}{\left[ageatBL\right]}_{j}+\beta_{7}{\left[malesex\right]}_{j}+\beta_{8}{\left[diseasedurationatBL\right]}_{j}+\sum_{k=1}^{16}\beta_{9.k}{\left[{\mathrm{centre}}_{k}\right]}_{j}+\varepsilon_{\mathrm{ij}},where\left(u_{0j},u_{1j}\right)∼N\left(0,\sum_{u}\right),and\varepsilon_{\mathrm{ij}}∼NID\left(0,\sigma^{2}\right)$$

$y_{\mathrm{ij}}=$ SPACE-MS variable at ${\mathrm{time}}_{i}$ for ${\mathrm{subject}}_{j}$ (please note that six longitudinal models were built, one for each SPACE-MS metric); BL = baseline; FU = follow-up; ${(\beta }_{0}+u_{0j})$ = mean (across subjects) intercept ($\beta_{0}$) plus subject-specific random component of the intercept ($u_{0j})$; that is, this model estimates one intercept for each subject; ${(\beta }_{1}+u_{1j})$ = mean (across subjects) regression coefficient for time ($\beta_{1}$) plus subject-specific random component of the slope for time ($u_{1j}$); that is, this model estimates one regression coefficient (or slope) for time for each subject; from $\beta_{2}$ to $\beta_{9}$ = regression coefficients for the rest of variables, which do not change over time and which do not have any subject-specific random component (i.e. these are fixed effects). This model assumes that the two random components ($u_{0j},u_{1j}$), given the covariates, follow a multivariate normal distribution, where $\sum_{u}$ is a 2 × 2 matrix: $\left(\begin{array}{cc} \sigma_{u00}^{2} & \sigma_{u10}\\ \sigma_{u01} & \sigma_{u11}^{2} \end{array}\right)$ which represents the covariance structure of the model (we allowed this to be *unstructured*); $\sigma_{u00}^{2}$ = between-subject variance of (the dependent variable) at the intercept (i.e. at time = 0); $\sigma_{u11}^{2}$ = between-subject variance of the slopes for time; $\sigma_{u01}=\sigma_{u10}=$covariance between slope and intercept (we allowed this to take any number). This model also assumed that (so-called *level-1*) residuals $\varepsilon_{\mathrm{ij}}$ were normally and identically distributed, with mean = 0 and variance = $\sigma^{2}$ .

As an exploratory analysis, other longitudinal models were also built where the variable ‘time’ was reparameterised as: 1) ‘disease duration’, given that disease duration also increases with increasing study follow-up, and considering that not all patients had the same disease duration at study onset (this model was adjusted for age); and as 2) ‘age’, given that age increases with increasing study follow-up, and not all patients had the same age at study baseline (this model was built with and without adjusting for disease duration). These two models gave us the possibility of covering a much wider range of the progression period than when we modelled the evolution of MRI parameters over the relatively short study follow-up period. The potential non-linear behaviour of SPACE-MS metrics over time was also explored, through the addition of quadratic terms for the time variable, in all longitudinal models.

Model assumptions were checked for all models. Statistical significance was set at 0.05. All the statistical analyses were carried out in Stata/SE 14.2.

## 4 Results

SPACE-MS was successfully implemented and applied to a cohort of 515 patients with progressive MS. The introduction of SPACE-MS metrics revealed characteristics of lesion distribution and location that are important for explaining concurrent disability and predict future disability accrual, beyond traditional metrics of lesion load and GM and WM volumes. Detailed results are reported here below.

### 4.1 Descriptive statistics and preliminary analyses

Clinical, demographic and MRI variables at baseline are shown in Table 1. Unadjusted baseline associations between SPACE-MS metrics and demographic, clinical, and MRI variables are shown in Table 2. This table also shows the correlations between the different SPACE-MS metrics. When age-, sex- and disease-duration-adjusted values of SPACE-MS metrics were compared across all 16 study centres, very similar values were observed (Fig. 4). Clinical measures at baseline and follow-up were highly correlated, as expected, given the relatively short follow-up of our cohort (Supplementary Table 1).

**Table 1:** Clinical, demographical and MRI characteristics at baseline.

	All patient Mean (SD)*	MS-SMART trial cohort Mean (SD)*	MS-STAT trial cohort Mean (SD)*	Observational cohort Mean (SD)*	p-value, MS-SMART vs MS-STAT^#^	p-value, MS-SMART vs observational cohort^#^	p-value, MS-STAT vs observational cohort^#^
**Clinical and demographical variables**							
Number of patients	515	356	126	33	–	–	–
Age in years	53.83 (7.25)	54.94 (6.90)	51.22 (7.03)	51.79 (8.78)	<0.001	0.0149	0.6935
Sex, N males (%)	172 (33.4%)	118 (33.2%)	41 (32.5%)	13 (39.4%)	0.901^&^	0.468^&^	0.459^&^
Disease duration in years	21.765 (9.677)	22.20 (9.85)	20.99 (8.74)	20.00 (11.12)	0.2247	0.2311	0.5873
EDSS score, median (range)	6.0 (3.0 to 8.5)	6.0 (4.0 to 6.5)	6.0 (4.0 to 7.0)	6.0 (3.0 to 8.5)	0.5948^£^	0.8918^£^	0.7609^£^
Inverse of TWT in 1/s	0.085 (0.045)	0.092 (0.047)	0.065 (0.034)	–	<0.001	–	–
Inverse of 9HPT in 1/s	0.034 (0.010)	0.034 (0.010)	0.034 (0.009)	–	0.8673	–	–
PASAT raw score	37.672 (14.973)	38.478 (15.058)	35.283 (14.518)	–	0.0432	–	–
SDMT raw score	44.008 (12.368)	44.218 (12.532)	–	41.53 (10.08)	–	0.2542	–

**Volumetric brain MRI variables (in mL)**							
T2 lesion load	16.060 (13.954)	13.087 (12.379)	23.874 (14.567)	18.291 (15.959)	0.003	0.770	0.041
NAWM volume	546.303 (75.573)	586.309 (44.737)	466.703 (42.841)	418.659 (52.993)	<0.001	<0.001	<0.001
CGM volume	728.529 (104.401)	790.007 (43.824)	594.371 (59.668)	577.555 (58.635)	<0.001	<0.001	0.174
DGM volume	40.973 (6.764)	44.591 (4.094)	32.703 (3.998)	33.519 (3.783)	<0.001	<0.001	0.076

**SPACE-MS metrics (dimensionless units unless otherwise specified)**							
NCI	0.481 (0.073)	0.480 (0.079)	0.491 (0.054)	0.454 (0.066)	0.153	0.715	<0.001
Maximum lesion NCI	1.140 (0.145)	1.120 (0.137)	1.223 (0.124)	1.047 (0.166)	<0.001	0.111	<0.001
MCI, in mm^2^	599.867 (107.241)	613.666 (108.274)	567.673 (94.529)	573.932 (113.476)	0.448	0.639	0.787
CAI	0.582 (0.107)	0.593 (0.113)	0.554 (0.082)	0.570 (0.117)	0.043	0.877	0.241
CPI	0.384 (0.134)	0.385 (0.133)	0.375 (0.133)	0.408 (0.143)	0.657	0.614	0.071
CSI	0.368 (0.121)	0.356 (0.124)	0.403 (0.100)	0.364 (0.130)	0.013	0.945	0.013

*unless otherwise specified; #: unadjusted comparisons, through independent samples *t*-test, unless otherwise specified; &: chi-square test; £: Mann-Whitney *U* test. *Abbreviations (in alphabetical order):* 9HPT: nine-hole peg test; CAI: covariance anisotropy index; CI: Confidence Interval; CPI: covariance planarity index; CSI: covariance sphericity index; EDSS: expanded disability status scale; GM: grey matter; Max: maximum; MCI: mean covariance index; NAWM: normal-appearing white matter; NCI: neuraxis caudality index; PASAT: paced auditory serial addition test (measured in number of correct answers); RC: regression coefficient; SD: standard deviation; SDMT: symbol digit modalities test (measured in number of correct answers); SPACE-MS: spatial patterns (of MS lesions) assessed through covariance estimations of lesional voxels; TWT: 25-foot timed walk test.

**Table 2:** Associations of SPACE-MS metrics with demographic, clinical and MRI variables^#^

	SPACE-MS^§^					
	NCI	Maximum lesion NCI	MCI	CAI	CPI	CSI
**Demographic variables**						
Age in years	−0.0909 **p = 0.0339**	−0.0414 p = 0.3351	0.0931 **p = 0.0299**	0.1778 **p < 0.0001**	0.1938 **p < 0.0001**	−0.2476 **p < 0.0001**
Sex (male)*	0.0485 p = 0.2575	−0.0033 p = 0.9378	0.2268 **p < 0.0001**	−0.0394 p = 0.3577	0.0090 p = 0.8343	0.0473 p = 0.2702

**Clinical variables**						
Disease duration in years	−0.1086 p = 0.4129	−0.0539 p = 0.6854	−0.0092 p = 0.9448	0.0140 p = 0.9165	0.2489 p = 0.0574	−0.1265 p = 0.3399
EDSS score*	−0.0266 p = 0.5372	0.1215 **p = 0.0046**	0.0729 p = 0.0902	−0.0690 p = 0.1088	−0.0058 p = 0.8921	0.0719 p = 0.0947
Inverse of TWT in 1/s	0.1002 **p = 0.0294**	−0.1559 **p = 0.0007**	0.0643 p = 0.1634	0.1365 **p = 0.0030**	−0.0170 p = 0.7128	−0.1161 **p = 0.0116**
Inverse of 9HPT in 1/s	−0.0048 0.9165	−0.1721 **p = 0.0002**	−0.1323 **p = 0.0037**	0.2330 **p < 0.0001**	−0.0137 p = 0.7645	−0.1922 **p < 0.0001**
PASAT raw score	0.1860 **p < 0.0001**	−0.0766 0.0949	0.0566 p = 0.2180	0.1724 **p = 0.0002**	−0.1151 **p = 0.0119**	−0.0886 0.0534
SDMT raw score	0.1167 **p = 0.0180**	−0.2399 **p < 0.0001**	−0.0013 p = 0.9786	0.2626 **p < 0.0001**	−0.0841 p = 0.0890	−0.1951 **p = 0.0001**

**MRI variables^+^**						
T2 lesion load	−0.2685 **p < 0.0001**	0.1846 **p < 0.0001**	0.0824 p = 0.0657	−0.2831 **p < 0.0001**	0.3141 **p < 0.0001**	0.0782 p = 0.0806
NAWM volume	0.1401 **p = 0.0017**	−0.1034 p = 0.0207	0.2047 **p < 0.0001**	0.1636 **p = 0.0002**	−0.1683 **p = 0.0002**	−0.0519 p = 0.2470
GM volume	0.1164 **p = 0.0092**	−0.0401 p = 0.3714	0.1423 **p = 0.0014**	0.0996 p = 0.0259	−0.2499 **p < 0.0001**	0.0514 p = 0.2509
CGM volume	0.1156 **p = 0.0097**	−0.0306 = 0.4951	0.1408 **p = 0.0016**	0.0906 p = 0.0429	−0.2437 **p < 0.0001**	0.0561 p = 0.2106
DGM volume	0.0937 p = 0.0363	−0.1426 **p = 0.0014**	0.1205 **p = 0.0070**	0.1796 **p < 0.0001**	−0.2541 **p < 0.0001**	−0.0187 p = 0.6774

**SPACE-MS metrics^+^**						
NCI	1	–	–	–	–	–
Maximum lesion NCI	0.1772 **p = 0.0001**	1	–	–	–	–
MCI	0.1648 **p = 0.0002**	0.1169 **p = 0.0089**	1	–	–	–
CAI	0.0641 p = 0.1526	−0.3348 **p < 0.0001**	0.1411 **p = 0.0016**	1	–	–
CPI	−0.2007 **p < 0.0001**	−0.1162 **p = 0.0093**	−0.2270 **p < 0.0001**	−0.1416 **p = 0.0015**	1	–
CSI	0.0634 p = 0.1571	0.3933 **p < 0.0001**	0.0025 p = 0.9563	−0.8186 **p < 0.0001**	−0.4368 **p < 0.0001**	1

#: Pearson’s correlation coefficient, unless otherwise specified; *: Spearman’s correlation coefficient; + for the correlations between SPACE-MS and other MRI variables, partial correlation coefficients adjusting for scanning centre are presented (instead of unadjusted Pearson’s correlation coefficients); §: only the strongest associations (p < 0.01) are highlighted. *Abbreviations (in alphabetical order):* 9HPT: nine-hole peg test; CAI: covariance anisotropy index; CI: Confidence Interval; CPI: covariance planarity index; CSI: covariance sphericity index; EDSS: expanded disability status scale; GM: grey matter; Max: maximum; MCI: mean covariance index; NAWM: normal-appearing white matter; NCI: neuraxis caudality index; PASAT: paced auditory serial addition test (measured in number of correct answers); RC: regression coefficient; SD: standard deviation; SDMT: symbol digit modalities test (measured in number of correct answers); SPACE-MS: spatial patterns (of MS lesions) assessed through covariance estimations of lesional voxels; TWT: 25-foot timed walk test.

**Fig. 4:**
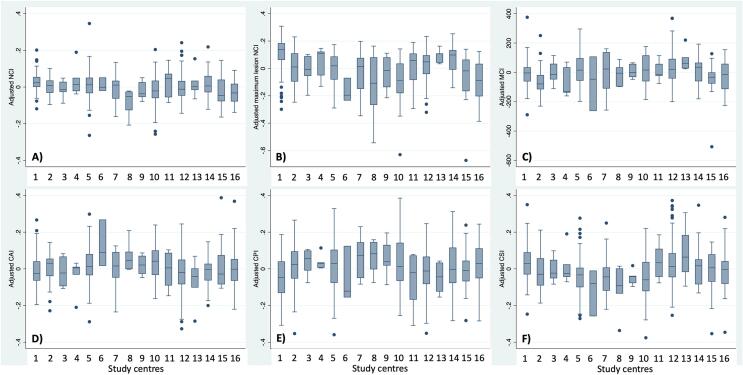
**Adjusted values of SPACE-MS metrics for all study centres.** This figure shows the age-, sex-, disease-duration-, and lesion-load-adjusted values of SPACE-MS metrics for all 16 study centres. Overall, such adjusted values of SPACE-MS metrics were very similar across centres. Importantly, though, the values shown in this figure have not been adjusted for disability measures, so the variability across centres due to differences in disability scores between centres has not been removed. *Abbreviations*: CAI: covariance anisotropy index; CPI: covariance planarity index; CSI: covariance sphericity index; NCI: neuraxis caudality index.

### 4.2 Explanation of disability measures at baseline

The best a priori models showed expected results, being a higher lesion volume at baseline the best overall predictor of worse disability, for all clinical outcomes (Table 3). Larger WM and GM volumes were associated with better clinical scores. Older age at study baseline was associated with better clinical scores and male sex had a less clear effect: it was significantly associated with better EDSS (p = 0.003), TWT (p = 0.004) and PASAT (p = 0.004) outcomes, whereas it was associated with worse NHPT performance (p < 0.001). Longer disease duration was associated with worse clinical outcomes (Table 3).

**Table 3:** Explanation of concurrent disability using SPACE-MS metrics.

	Clinical variable at baseline^#^ (dependent variable)				
	EDSS score	Inverse of TWT	Inverse of 9HPT	PASAT score	SDMT score
**Best a priori models**					
**Model R^2^**	0.1156	0.2071	0.2193	0.2102	0.3414
Main predictors (below)					
**Lesion load at baseline in mL**RC (95%CI), p-value	0.009 (0.003 to 0.014), **p = 0.002**	−0.0004 (−0.0007 to −0.0001), **p = 0.009**	−0.0002 (−0.0003 to −0.00015), **p < 0.001**	−0.371 (-0.471 to −0.271), **p < 0.001**	−0.405 (-0.492 to −0.317), **p < 0.001**
**WM volume at baseline in mL**RC (95%CI), p-value	−0.001 (−0.004 to 0.002), p = 0.439	0.0001 (−0.00001 to 0.0003), p = 0.077	0.00004 (0.00001 to 0.00007), **p = 0.006**	0.003 (−0.041 to 0.048), p = 0.885	0.087 (0.049 to 0.125), **p < 0.001**
**GM volume at baseline in mL**RC (95%CI), p-value	−0.0004 (−0.003 to 0.002), p = 0.752	−0.00003 (−0.0001 to 0.0001), p = 0.670	6.41•10^-06^ (−0.00002 to 0.00003), p = 0.635	0.041 (0.001 to 0.081), **p = 0.042**	−0.008 (−0.045 to 0.030), p = 0.683
**Age at baseline in years**RC (95%CI), p-value	0.006 (−0.005 to 0.017), p = 0.261	−0.0004 (−0.001 to 0.0002), p = 0.178	0.0002 (0.0001 to 0.0003), **p = 0.004**	0.272 (0.074 to 0.469), **p = 0.007**	0.048 (-0.123 to 0.219), p = 0.581
**Male sex**RC (95%CI), p-value	−0.222 (-0.370 to −0.073), **p = 0.003**	0.012 (0.004 to 0.021), **p = 0.004**	−0.003 (−0.005 to −0.001), **p < 0.001**	3.974 (1.292 to 6.655), **p = 0.004**	−0.938 (-3.275 to 1.399), p = 0.430
**Disease duration at baseline in years**RC (95%CI), p-value	0.009 (0.001 to 0.017), **p = 0.028**	−0.0006 (−0.0011 to −0.0002), **p = 0.007**	−0.0001 (−0.00020 to −8.33•10^-06^), **p = 0.033**	−0.011 (-0.156 to 0.134), p = 0.879	−0.040 (-0.160 to 0.081), p = 0.514

**SPACE-MS models of concurrent disability^&^**					
Baseline SPACE-MS metrics (below)^$^					
**NCI**RC (95%CI), p-value *Model R^2^ (%I)*	−0.135 (−1.151 to 0.880), p = 0.793 *0.1158 (0.2%)*	0.031 (−0.025 to 0.086), p = 0.276 *0.2092 (1.0%)*	−0.016 (−0.028 to −0.004), p = 0.010 ***0.2308 (5.2%)***	20.106 (2.061 to 38.152), p = 0.029 ***0.2185 (3.9%)***	−7.103 (-21.637 to 7.430), p = 0.337 *0.3431 (0.5%)*
**Maximum lesion NCI**RC (95%CI), p-value *Model R^2^ (%I)*	0.593 (0.046 to 1.140), p = 0.034 ***0.1238 (7.1%)***	−0.030 (−0.061 to 0.001), p = 0.058 *0.2135 (3.1%)*	−0.009 (−0.015 to −0.002), p = 0.011 ***0.2305 (5.1%)***	−0.423 (−10.478 to 9.632), p = 0.934 *0.2102 (0%)*	−10.978 (−19.161 to −2.796), p = 0.009 ***0.3540 (3.7%)***
**MCI**RC (95%CI), p-value *Model R^2^ (%I)*	0.0004 (−0.0003 to 0.001), p = 0.245 *0.1181 (2.2%)*	−7.16•10^-06^(−0.00005 to 0.00003), p = 0.712 *0.2073 (0.1%)*	−0.00001(−0.00002 to−3.95•10^-06^), p = 0.004 ***0.2336 (6.5%)***	−0.001 (−0.014 to 0.011), p = 0.857 *0.2102 (0%)*	0.0003 (−0.010 to 0.011), p = 0.949 *0.3414 (0%)*
**CAI**RC (95%CI), p-value *Model R^2^ (%I)*	−0.780 (−1.484 to −0.076), p = 0.030 ***0.1242 (7.4%)***	0.049 (0.011 to 0.088), p = 0.013 ***0.2181 (5.3%)***	0.012 (0.003 to 0.020), p = 0.006 ***0.2323 (5.9%)***	10.248 (−2.599 to 23.095), p = 0.118 *0.2145 (2.0%)*	14.667 (4.500 to 24.834), p = 0.005 ***0.3559 (4.2%)***
**CPI**RC (95%CI), p-value *Model R^2^ (%I)*	−0.519 (−1.088 to 0.049), p = 0.073 *0.1215 (5.1%)*	0.024 (−0.007 to 0.055), p = 0.131 *0.2111 (1.9%)*	0.008 (0.001 to 0.015), p = 0.023 ***0.2281 (4.0%)***	1.196 (−9.090 to 11.483), p = 0.819 *0.2103 (0.1%)*	6.921 (−1.65248 15.49546), p = 0.113 *0.3460 (1.3%)*
**CSI**RC (95%CI), p-value *Model R^2^ (%I)*	0.960 (0.343 to 1.578), p = 0.002 ***0.1323 (14.4%)***	−0.053 (−0.087 to −0.019), p = 0.002 ***0.2237 (8.0%)***	−0.013 (−0.021 to −0.006), p < 0.001 ***0.2408 (9.8%)***	−8.953 (−20.189 to 2.283), p = 0.118 *0.2145 (2.0%)*	−15.562 (−24.667 to −6.458), p = 0.001 ***0.3616 (5.9%)***

**Best SPACE-MS model of concurrent disability**					
**Best SPACE-MS metric(s) at baseline**RC (95%CI), p-value *Model R^2^ (%I)*	CSI: 0.960(0.343 to 1.578), **p = 0.002** ***0.1323 (14.4%)***	CSI: −0.053(−0.087 to −0.019), **p = 0.002** ***0.2237 (8.0%)***	CSI: −0.013(−0.020 to −0.005), **p = 0.001** NCI: −0.014(−0.026 to −0.002), **p = 0.019** ***0.2500 (14.0%)***	NCI: 20.106 (2.061 to 38.152), **p = 0.029** ***0.2185 (3.9%)***	CSI: −15.562(−24.667 to −6.458), **p = 0.001** ***0.3616 (5.9%)***

The ‘best a priori model’ included, as covariates, baseline lesion volume, baseline WM volume, baseline GM volume, age in years at baseline, male sex, disease duration at baseline, and study centre. Of note, the regression coefficients for ‘study centre’ are not shown (for simplicity). The ‘SPACE-MS models’ included all the variables of the ‘best a priori model’ plus one SPACE-MS metric at a time. See methods for full details. #: the EDSS score is measured in EDSS score units; the inverse of TWT and the inverse of 9HPT, in 1/s; and the PASAT and SDMT scores, in number of correct answers; &: all ‘SPACE-MS models’ included, as covariates, the variables included in the ‘best a priori models’. However, for simplicity, only the RC (95%CI) of the SPACE-MS metric is shown; $: all SPACE-MS metrics are measured in dimensionless units except for MCI, which is measured in mm^2^. Significant results are highlighted. *Abbreviations (in alphabetical order):* %I: percentage of model improvement based on R^2^; 9HPT: nine-hole peg test; CAI: covariance anisotropy index; CI: Confidence Interval; CPI: covariance planarity index; CSI: covariance sphericity index; EDSS: expanded disability status scale; GM: grey matter; Max: maximum; MCI: mean covariance index; NAWM: normal-appearing white matter; NCI: neuraxis caudality index; PASAT: paced auditory serial addition test (measured in number of correct answers); RC: regression coefficient; SD: standard deviation; SDMT: symbol digit modalities test (measured in number of correct answers); SPACE-MS: spatial patterns (of MS lesions) assessed through covariance estimations of lesional voxels; TWT: 25-foot timed walk test.

**Fig. 5:**
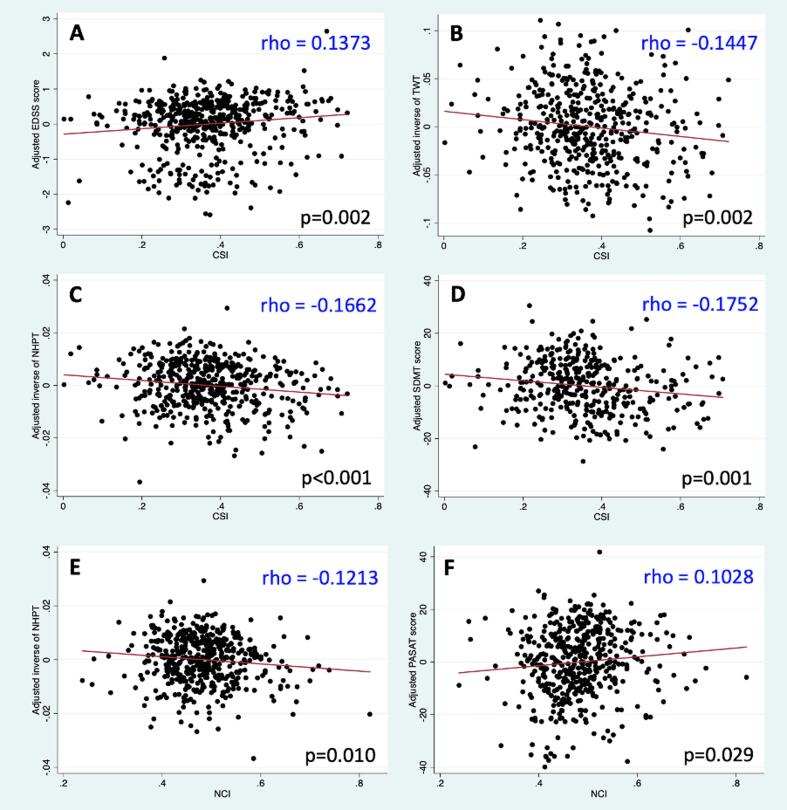
**Baseline associations between spatial distribution metrics and clinical variables.** This figure shows the main associations between spatial distribution metrics and clinical variables at baseline, after adjusting for all relevant confounders. For graphical purposes only, the y-axis in this figure indicates the values of the clinical variable at baseline (dependent variable) after having been adjusted for all the covariates in the corresponding ‘best a priori model’ (as indicated in the methods and Table 3). *Abbreviations in alphabetical order*: CSI: covariance sphericity index; EDSS: expanded disability status scale; NCI: neuraxis caudality index; NHPT: nine-hole peg test; PASAT: paced auditory serial addition test; SDMT: symbol digit modalities test; TWT: 25-foot timed walk test.

When SPACE-MS metrics were added individually to the five best a priori models, several improvements in model performance were observed: higher caudality of the whole-brain lesion mask and/or higher caudality of the most caudal brain lesion were associated with worse motor disability as measured by the EDSS (*maximum lesion NCI*, p = 0.034) or NHPT (*NCI*, p = 0.010; *maximum lesion NCI*, p = 0.011). Higher caudality of the whole-brain lesion mask was also associated with better PASAT scores (p = 0.029). Instead, patients whose most caudal lesion was more caudal performed worse on the SDMT (p = 0.009). Patients whose whole-brain lesion masks showed greater spreading (i.e. higher MCI) or this spreading was more isotropic (i.e. higher CSI and/or smaller CAI) showed significantly greater disability levels for all clinical measures except for PASAT. Fig. 5 shows the significant (adjusted) associations of CSI and NCI with clinical variables.

When all SPACE-MS metrics were included together in the model as predictors (together with the predictors of the best a priori models), (higher) CSI appeared as the best overall predictor of (worse) performance on the EDSS (p = 0.002), TWT (p = 0.002), and SDMT (p = 0.001). For the NHPT, both higher CSI (p = 0.001) and higher NCI (p = 0.019) independently predicted worse performance. Finally, (higher) NCI appeared as the best predictor of (better) PASAT performance (p = 0.029).

Similar predictions were observed when the ‘best *a priori* models’ were built following a backward elimination strategy (Supplementary Table 2).

### 4.3 Prediction of future disability

The best a priori models showed that the clinical score at baseline was the strongest predictor of future disability for all clinical scores. Larger T2 lesion volumes were also predictive of worse outcome. The role of the rest of the a priori predictors was much less clear (Table 4).

**Table 4:** Prediction of future disability using SPACE-MS metrics.

	Clinical variable at follow-up^#^ (dependent variable)				
	EDSS score	Inverse of TWT	Inverse of 9HPT	PASAT score	SDMT score
**Best a priori models**					
**Model R^2^**	0.6205	0.5607	0.7528	0.6522	0.8063
Main predictors (below)					
**Lesion load at baseline in mL**RC (95%CI), p-value	3.22•10^-06^(-1.91•10^-06^ to 8.35•10^-06^), p = 0.219	−3.66•10^-07^(-6.50•10^-07^ to−8.20•10^-08^), **p = 0.012**	−3.56•10^-08^(-8.31•10^-08^ to 1.18•10^-08^), p = 0.140	−0.00025(−0.00033 to−0.00017), **p < 0.001**	−0.00019(−0.00026 to−0.00013), **p < 0.001**
**WM volume at baseline in mL**RC (95%CI), p-value	2.65•10^-08^(-2.21•10^-06^ to 2.27•10^-06^), p = 0.981	−4.83•10^-08^(-1.70•10^-07^ to 7.37•10^-08^), p = 0.437	−3.96•10^-09^(-2.44•10^-08^ to 1.64•10^-08^), p = 0.703	0.00004(2.28•10^-06^ to 0.00007), **p = 0.036**	5.14•10^-06^(−0.00002 to 0.00003), p = 0.686
**GM volume at baseline in mL** RC (95%CI), p-value	−4.49•10^-07^(−2.42•10^-06^ to 1.52•10^-06^), p = 0.654	5.07•10^-08^(−5.87•10^-08^ to 1.60•10^-07^), p = 0.363	1.28•10^-08^(−5.13•10^-09^ to 3.07•10^-08^), p = 0.161	4.95•10^-06^(−0.00002 to 0.00003), p = 0.743	9.73•10^-06^(−0.00002 to 0.00003), p = 0.445
**Age at baseline in years** RC (95%CI), p-value	−0.008(−0.017 to 0.002), p = 0.116	0.0004(−0.0001 to 0.001), p = 0.144	0.00006(−0.00003 to 0.00015), p = 0.177	0.168 (0.022 to 0.314), **p = 0.024**	0.090 (−0.022 to 0.202), p = 0.113
**Male sex** RC (95%CI), p-value	0.014 (−0.119 to 0.146), p = 0.840	0.001 (−0.007 to 0.008), p = 0.883	−0.0002 (−0.001 to 0.001), p = 0.734	2.177 (0.179 to 4.175), **p = 0.033**	−0.541 (-2.096 to 1.013), p = 0.494
**Disease duration at baseline in years** RC (95%CI), p-value	−0.007 (−0.015 to −0.0003), **p = 0.042**	0.00049 (0.00009 to 0.00089), **p = 0.017**	0.00003(−0.00004 to 0.00009), p = 0.403	−0.039 (−0.145 to 0.068), p = 0.474	−0.042 (−0.120 to 0.036), p = 0.287
**Clinical variable at baseline** RC (95%CI), p-value	1.027 (0.950 to 1.105), **p < 0.001**	0.860 (0.777 to 0.943), **p < 0.001**	0.961 (0.899 to 1.023), **p < 0.001**	0.720 (0.651 to 0.789), **p < 0.001**	0.843 (0.776 to 0.911), **p < 0.000**

**SPACE-MS models of future disability^&^**					
Baseline SPACE-MS metrics (below)^$^					
**NCI**RC (95%CI), p-value *Model R^2^ (%I)*	1.114 (0.217 to 2.012), **p = 0.015** ***0.6253 (0.8%)***	−0.044 (−0.094 to 0.005), p = 0.079 *0.5641 (0.6%)*	0.001 (−0.008 to 0.009), p = 0.902 *0.7528 (0%)*	4.084 (-9.477 to 17.644), p = 0.554 *0.6525 (0.1%)*	−5.118 (-14.567 to 4.331), p = 0.287 *0.8070 (0.1%)*
**Maximum lesion NCI**RC (95%CI), p-value *Model R^2^ (%I)*	0.453 (−0.033 to 0.939), p = 0.068 *0.6232 (0.4%)*	−0.023 (−0.051 to 0.005), p = 0.109 *0.5635 (0.5%)*	−0.004 (−0.008 to 0.001), p = 0.118 *0.7542 (0.2%)*	1.659 (-5.825 to 9.143), p = 0.6603 *0.6523 (0.02%)*	−8.669 (-14.040 to −3.299), **p = 0.002** ***0.8122 (0.7%)***
**MCI**RC (95%CI), p-value *Model R^2^ (%I)*	−0.0002(−0.0008 to 0.0004), p = 0.503 *0.6209 (0.1%)*	0.00001(−0.00002 to 0.00004), p = 0.523 *0.5611 (0.1%)*	−5.39•10−0^7^(-6.19•10−0^6^ to 5.11•10−0^6^), p = 0.851 *0.7528 (0%)*	0.004 (−0.006 to 0.013), p = 0.439 *0.6527 (0.1%)*	−0.001 (−0.008 to 0.005), p = 0.664 *0.8064 (0.01%)*
**CAI**RC (95%CI), p-value *Model R^2^ (%I)*	−0.523 (-1.149 to 0.103), p = 0.101 *0.6227 (0.4%)*	0.017 (−0.019 to 0.052), p = 0.352 *0.5616 (0.2%)*	0.007 (0.001 to 0.013), **p = 0.018** ***0.7560 (0.4%)***	−0.188 (-9.802 to 9.427), p = 0.969 *0.6522 (0%)*	7.970 (1.237 to 14.703), **p = 0.020** ***0.8095 (0.4%)***
**CPI**RC (95%CI), p-value *Model R^2^ (%I)*	−0.172 (−0.686 to 0.343), p = 0.513 *0.6208 (0.1%)*	0.023 (−0.006 to 0.052), p = 0.125 *0.5633 (0.5%)*	0.004 (−0.001 to 0.008), p = 0.123 *0.7542 (0.2%)*	−4.428 (-12.093 to 3.237), p = 0.257 *0.6532 (0.2%)*	3.198 (-2.489 to 8.885), p = 0.269 *0.8071 (0.1%)*
**CSI**RC (95%CI), p-value *Model R^2^ (%I)*	0.498 (−0.057 to 1.054), p = 0.079 *0.6230 (0.4%)*	−0.028 (−0.060 to 0.004), p = 0.082 *0.5640 (0.6%)*	−0.008 (−0.013 to −0.003), **p = 0.002** ***0.7585 (0.8%)***	3.640 (-4.759 to 12.039), p = 0.395 *0.6528 (0.1%)*	−8.317 (-14.378 to −2.257), **p = 0.007** ***0.8106 (0.5%)***

**Best SPACE-MS model of future disability**					
**Best SPACE-MS metric(s) at baseline**RC (95%CI), p-value *Model R^2^ (%I)*	NCI: 1.114 (0.217 to 2.012), **p = 0.015** ***0.6253 (0.8%)***	NCI: −0.044(−0.094 to 0.005), p = 0.079 *0.5641 (0.6%)*	CSI: −0.008(−0.013 to−0.003), **p = 0.002** ***0.7585 (0.8%)***	CPI: −4.428(-12.093 to 3.237), p = 0.257 *0.6532 (0.2%)*	Maximum lesion NCI: −8.669 (-14.040 to −3.299), **p = 0.002** ***0.8122 (0.7%)***

The ‘best a priori model’ included, as covariates, baseline lesion volume, baseline WM volume, baseline GM volume, age in years at baseline, male sex, disease duration at baseline, centre, and clinical variable at baseline. Of note, the regression coefficients for ‘study centre’ are not shown (for simplicity). The ‘SPACE-MS models’ included all the variables of the ‘best a priori model’ plus one SPACE-MS metric at baseline at a time. See methods for full details. #: the EDSS score is measured in EDSS score units; the inverse of TWT and the inverse of 9HPT, in 1/s; and the PASAT and SDMT scores, in number of correct answers; &: all ‘SPACE-MS models’ included, as covariates, the variables included in the ‘best a priori models’. However, for simplicity, the RC (95% CI) of all these extra variables are not shown; $: all spatial distribution metrics are measured in dimensionless units except for MCI, which is measured in mm^2^. Significant results are highlighted. *Abbreviations (in alphabetical order):* %I: percentage of model improvement based on R^2^; 9HPT: nine-hole peg test; CAI: covariance anisotropy index; CI: Confidence Interval; CPI: covariance planarity index; CSI: covariance sphericity index; EDSS: expanded disability status scale; GM: grey matter; Max: maximum; MCI: mean covariance index; NAWM: normal-appearing white matter; NCI: neuraxis caudality index; PASAT: paced auditory serial addition test (measured in number of correct answers); RC: regression coefficient; SD: standard deviation; SDMT: symbol digit modalities test (measured in number of correct answers); SPACE-MS: spatial patterns (of MS lesions) assessed through covariance estimations of lesional voxels; TWT: 25-foot timed walk test.

When SPACE-MS metrics were added individually to these five best a priori models, modest but significant improvements in model performance were observed for prediction of EDSS, NHPT and SDMT at follow-up. No significant improvements were observed for TWT or PASAT. In general, having a more caudal whole-brain lesion mask or lowermost brain lesion implied worse disability scores at follow-up as measured by the EDSS (p = 0.015) or the SDMT (p = 0.002), respectively. Additionally, having a more isotropic distribution of the lesions in the brain at baseline (i.e. higher CSI and/or lower CAI) also implied worse disability at follow-up, as measured by the NHPT (CAI, p = 0.018; CSI: p = 0.002) and the SDMT (CAI: p = 0.020; CSI: p = 0.007).

When all SPACE-MS metrics were added together to the best a priori model for each clinical outcome, the best SPACE-MS predictors were NCI (for EDSS, p = 0.015), CSI (for NHPT, p = 0.002), and maximum lesion NCI (for SDMT, p = 0.002) (Table 4).

Similar predictions were observed when the ‘best *a priori* models’ were built following a backward elimination strategy (Supplementary Table 3).

### 4.4 Dynamic changes in spatial distribution metrics

Over time, there was a significant decrease in NCI of −0.0007 units/year (95% Confidence Interval [95%CI]) −0.0013 to −0.0002), p = 0.012, indicating that the whole-brain masks became more cranial as time went on. Instead, maximum lesion NCI significantly increased during the follow-up by 0.0011 units/year (95%CI 0.0002 to 0.0021), p = 0.017, indicating that the most caudal brain lesion tended to be more caudal as time went on. Additionally, the isotropy of the whole-brain lesion mask tended to increase as time went on, as reflected by a significant decrease in CAI by −0.0008 units/year −0.0014 to −0.0001), p = 0.018. No significant changes over time were observed in MCI (Regression Coefficient [RC] = 0.2854 mm^2^/year [95%CI: −0.5238 to 1.0945], p = 0.489), CPI (RC = 0.0007 units/year [−0.0005 to 0.0018], p = 0.256) or CSI (RC = 0.0005 [−0.0004 to 0.0013], p = 0.269).

Alternative models to quantify changes in SPACE-MS metrics with time-derived variables different from ‘(within-study) follow-up time’ did not materially provide different results. However, these models revealed a non-linear behaviour of the dynamic changes for NCI and CAI, which could not be captured with the original longitudinal models built, and which suggested a trend to plateau as age or disease duration increased. These models are shown in detail in Supplementary Table 4.

As expected, total lesion load significantly increased over time by 117.845 µL/year (95%CI: 84.517 to 151.173), p < 0.001.

### 4.5 Sensitivity and further exploratory analyses

As an exploratory analysis, individual-lesion (lesion-wise) metrics were computed and the variation of lesion shapes across the brain was visually assessed. Whereas lesion-wise NCI increased as the individual lesions became more caudal, as expected, there was not a clear pattern in the distribution of lesion shapes across the brain (Supplementary Fig. 1). When we assessed the relationship between lesion-wise and whole-brain SPACE-MS metrics, no strong correlations were observed, except for a moderate-strong correlation between whole-brain NCI and mean lesion-wise NCI (Supplementary Table 5).

Regarding the models built with the MNI-space SPACE-MS metrics as part of a sensitivity analysis, very similar results to those obtained with native-space (3DT1) metrics were obtained (Supplementary Tables 6 and 7).

## 5 Discussion

### 5.1 Summary and key findings

Here we presented SPACE-MS, a novel methodological approach to quantitatively assess aspects of WM lesion spatial distribution. Practically, such features can automatically be extracted from conventional structural MRI through our approach and could therefore be readily applied in clinical studies and trials. In our study in progressive MS, we found that spatial distributional features of brain lesions, when added to conventional predictive models of disability, significantly improved clinical outcome prediction. In particular, the presence of lesions in lower parts of the brain, and a more isotropic disposition of lesions emerged as particularly harmful features.

### 5.2 Caudality of lesions

At baseline, the location of lesions in lower parts of the brain, either referring to the whole-brain lesion mask or the lowermost brain lesion, was associated with greater motor disability, at baseline and over time, independently of lesion load and WM and GM volumes. The models based on conventional predictors of motor disability (e.g. EDSS) significantly improved after adding information on the caudality of lesions. This is the first time that a continuous metric enabling the quantitative assessment of lesion caudality is proposed. Interestingly, since the advent of the MRI to diagnose and monitor MS (Paty et al., 1988), numerous authors have shown that lesions affecting the infratentorial brain (Tintore et al., 2010, Chung et al., 2020, Kerbrat et al., 2020) or the spinal cord (Eden et al., 2019, Brownlee et al., 2019, Kerbrat et al., 2020) entail a particularly bad prognosis. However, this conventional information on lesion location is qualitative and requires visual inspection of the images. Thus, a major strength of our study is that the metrics we use to describe lesion caudality are fully automatic and objective, without requiring any expert neuro-radiological input.

A possible explanation for the strong association between higher NCI and greater motor disability could be that the impact of lesions is higher as the axonal density of the lesional tissue increases, as happens in more caudal parts of the WM tracts (Groeschel et al., 2016) and the cerebellum (Mitchell et al., 2019). This would be in line with a higher damaging effect of spinal cord lesions than that of infratentorial ones (Eden et al., 2019, Kerbrat et al., 2020), although this has not been studied and deserves future investigations. The association between lesion caudality and cognitive function, though, is not that clear: whereas a higher caudality of the whole-brain lesion mask implied a better cognitive function as measured by the PASAT, having a lowermost brain lesion more caudal was associated with worse cognitive function as measured by the SDMT. It is possible that patients with more caudal whole-brain lesion masks have fewer cortical/juxtacortical lesions, which have been associated with worse cognition (Cocozza et al., 2020), and this may have been the main determinant of PASAT performance. Instead, for the SDMT, other factors may have been involved. For instance, SDMT is known to be affected by visual impairment, and brainstem lesions can contribute to this (Smith, 2007). Another factor could be the presence of retrograde *trans*-synaptic degeneration (Haider et al., 2016) in the context of damage of long-range connections (Giovannoni et al., 2017), relevant for cognitive function in MS (Meijer et al., 2020). This would be in line with the observed progressive brain cortical atrophy following spinal cord injury (Ziegler et al., 2018).

A final note on lesion caudality is that, as time went on, there was a significant decrease in NCI together with a significant increase in maximum lesion NCI. This indicates that, in progressive MS, there is a cranial shift of the CoM of the whole-brain mask, in line with previous studies showing that in progressive MS, new lesions mainly tend to appear in the supratentorial brain (Gaetano et al., 2020).

Yet, our results also indicate that patients may tend to have their lowermost brain lesion more caudal as time goes by. Of note, these results were obtained after adjusting for baseline lesion load and changes in lesion load between baseline and follow-up, indicating a certain degree of independence between changes in lesion spatial distribution and crude changes in the amount or volume of lesions.

### 5.3 Spatial variability of lesional voxels

This is the first time that greater spatial variability of lesions is presented as a potential poor prognostic factor in MS. A greater amount of spatial variability of lesional voxels and, more importantly, a more isotropic distribution of such lesional voxels in the brain were associated with worse concurrent and future motor and cognitive disability. Interestingly, among all spatial distributional metrics, CSI appeared as the best predictor of concurrent disability for most clinical domains (together with NCI, for the NHPT), and the best predictor of future motor disability as measured by the NHPT. We speculate that more widespread distributions of lesions may reflect greater dissemination of demyelinating changes in the brain tissue. Also, greater dispersion might imply a greater number of WM tracts affected, therefore having detrimental consequences for the efficiency of the whole-brain network, which has been associated with worse disability outcomes on a number of occasions (Charalambous et al., 2019, Solana et al., 2018, Tur et al., 2020). Of note, the presence of juxtacortical/cortical lesions may also contribute to a higher dispersion of brain lesions. Thus, the consistent and strong association between higher CSI (or lower CAI) and worse disability might also be at least partly explained by the presence of a higher number of juxtacortical/cortical lesions among those patients with a greater dispersion of lesions. In this study we did not perform a formal analysis of cortical lesions. Nonetheless, when we adjusted our models for cortical GM volume (data not shown), whose decrease has been associated with the presence of juxtacortical/cortical lesions (Magliozzi et al., 2018), our results did not change. Future research combining the assessment of lesion spatial distribution, diffusion-based connectivity, cortical lesion detection, and quantitative MRI characterising those tissue microstructural changes that occur in MS, will provide further insights into the mechanisms linking more widespread lesion distributions and higher disability.

Remarkably, as time went by, lesional voxels tended to distribute more isotropically in the brain, as reflected by a significant decrease in CAI, after adjusting for covariates. This is the first time that this is reported in a quantitative way and may reflect the expansion of the demyelinating changes occurring in progressive MS. Importantly, SPACE-MS metrics may be explored as potential surrogate markers of disease progression for their use in clinical trials or help us understand the pathogenic mechanisms underpinning irreversible accumulation of disability. Future studies assessing longitudinal changes in lesion spatial distribution features and quantitative MRI metrics are warranted.

### 5.4 Methodological considerations

Some potential methodological considerations follow. First, in this study we defined the neuroaxis as the line from the primary motor cortex to the brainstem as a mean to calculate distribution of lesions through the brain. This was chosen because of the known motor impairment in MS and also because of existing studies reporting that infratentorial lesions seem to have a greater weight on disability (Tintore et al., 2010, Chung et al., 2020, Brownlee et al., 2019, Kerbrat et al., 2020). This is a choice, though, and future studies could choose a different definition of the neuroaxis or to study a different system.

Another aspect of our study is that we applied the SPACE-MS technique to MS data using a relatively homogeneous cohort, with high-quality clinical and MRI data, which might not be representative of routine practice. Nonetheless, we included participants from three different cohorts belonging to different centres, and therefore scanned on different scanners with different magnetic field strengths and imaging protocols. This offered a realistic portrait of real-world variability in terms of MRI hardware, software and protocols that could be encountered in hospital settings. Additionally, age-, sex-, disease-duration-, and lesion-load-adjusted mean values of SPACE-MS metrics were very similar across study centres, suggesting that these metrics are robust to variations in MRI acquisition protocols (Fig. 4).

In our study, lesions were segmented on T2-weighted images with anisotropic voxels (1 × 1 × 3 mm^3^). As 3D FLAIR is becoming more commonly acquired, in addition to conventional T2-weighed clinical scans, for MS lesion detection, with isotropic 1 mm^3^ resolution, it will be interesting to assess whether whole-brain and individual lesion metrics are affected by the anisotropic resolution of such T2-weighed scans. Of note, our study used semi-automated lesion segmentation, which may not be feasible in clinical practice. However, there are now fully automated lesion segmentation methods that can generate lesion masks that could be processed using this spatial distribution pipeline (Valverde et al., 2017).

Importantly, in our study we decided to compute the SPACE-MS metrics on native (3DT1) space instead of a common, unbiased space such as the MNI space. We made this decision to avoid the application of any non-linear co-registrations of our images which could alter the spatial distribution of lesions, which was the focus of our study. However, in a subset of patients, we also applied the SPACE-MS method to whole-brain lesion masks co-registered to the MNI brain. The overall results did not materially change, supporting the robustness of these metrics. Yet future studies exploring the convenience of using a common space (rather than a native one) for these types of analyses are needed.

In our study, given the relatively short follow-up of our patients and the absence of other, non-MRI metrics that could dynamically change over time, we did not build comprehensive disease progression models aimed at capturing an accurate picture of the temporal evolution of this condition (Jedynak et al., 2012, Donohue et al., 2014). Additionally, our application study focused on secondary progressive MS subjects, but also included a small cohort of primary progressive MS, given the increasing evidence that primary and secondary progressive MS share a number of pathogenetic features (Correale et al., 2016). For this analysis, therefore, a more traditional statistical approach provided the right tool for assessing the potential of SPACE-MS. Future studies are needed to investigate the role of spatial distributional metrics in a more heterogeneous group of subjects including other MS phenotypes, such as relapsing-remitting MS, and over longer periods of time. Another potential limitation of our data may be that, mainly due to the relatively short follow-up period of time between study baseline and the last time point, baseline clinical metrics were highly correlated with follow-up ones (see Supplementary Table 1), which decreased the ability of SPACE-MS metrics at baseline to predict clinical outcome at follow-up.

An additional note on our SPACE-MS metrics is that, when we added all SPACE-MS metrics at once in the predictive models of concurrent and future disability, we could see that there was frequently only one surviving predictor for a given clinical variable, suggesting a high level of collinearity. However, the ‘surviving’ predictor was not always the same, indicating that these metrics provided somehow complementary information. Furthermore, for the prediction of concurrent 9HPT score, both the CSI and the NCI appeared as independent predictors. These considerations support that both aspects of the distribution of lesions, i.e. their global spreading in the brain, measured through CSI, and their caudality, measured through NCI and maximum lesion NCI, play complementary roles when explaining or predicting disability.

Given the exploratory nature of this research, and considering the likely interdependencies between SPACE-MS metrics, no adjustment for multiple comparisons was performed.

In terms of future development of SPACE-MS, we believe that it will be important to include spinal cord lesions. We understand that spinal cord pathology is crucial to the development of disability progression and that having this type of data would have made our predictive models much more robust. Nonetheless, our aim, rather than building a very accurate predictive model of disability, was to understand whether information on the spatial distribution of lesions could add relevant information to those statistical models based on conventional metrics of lesion load and atrophy. Future studies accounting for spinal cord data are therefore warranted. Future developments of SPACE-MS also include its application to other neurological conditions characterised by WM lesions, such as primary or secondary CNS vasculitis, or SVD, where greater lesion volumes usually correlate with worse clinical outcome (Cannerfelt et al., 2018, Pantoni, 2010). In this context, the study of individual-lesion shapes, expanding the exploratory analyses performed here (Supplementary Fig. 1 and Supplementary Table 5) may provide very insightful information on the mechanisms of lesion formation in these conditions. More broadly, the potential of SPACE-MS can also be explored in other, non-neurological conditions characterised by visible lesions in any given organ outside the brain. For instance, in cancer research, the spatial characterisation of tumoral lesions has also started to emerge as a promising tool, in the context of radiomics, to predict clinical outcome (Limkin et al., 2019, van Griethuysen et al., 2017, Ligero et al., 2021).

## 6 Conclusions

With this study we introduced novel fully-automated metrics characterising clinically-relevant aspects of the spatial distribution of brain lesions. The metrics have been demonstrated and tested in a large longitudinal progressive MS cohort. We showed that location of lesions in lower parts of the brain, where neurite density is particularly high, and a greater spatial spreading of lesions, possibly reflecting a higher number of WM tracts involved, are relevant features for clinical deterioration in progressive MS, independent of brain lesion volume and atrophy. We therefore believe that their characterisation, which can be done using routinely acquired anatomical scans, may help achieve accurate predictions in the clinic, essential to the design of individualised therapeutic approaches. Of note, the usefulness of the SPACE-MS approach may be explored in other conditions, such as CNS vasculitis or SVD, also characterised by the presence of brain WM lesions and where greater lesion volumes have been associated with worse clinical outcomes, as happens in MS. (Cannerfelt et al., 2018, Pantoni, 2010)

## 7 Data and code availability statement

The code to run SPACE-MS is made freely available online (permanent link: https://github.com/carmentur/SPACE-MS). Researchers interested in accessing the data of this study can contact Prof Claudia Gandini Wheeler-Kingshott (c.wheeler-kingshott@ucl.ac.uk). A data sharing agreement enabling non-commercial research use will be stipulated. The scripts written to process the MRI scans and perform statistical analysis will be shared upon request (contact: Carmen Tur, c.tur@ucl.ac.uk, ctur@cem-cat.org).

### CRediT authorship contribution statement

**Carmen Tur:** Conceptualization, Data curation, Formal analysis, Funding acquisition, Investigation, Methodology, Project administration, Software, Visualization, Writing – original draft, Writing – review & editing. **Francesco Grussu:** Conceptualization, Methodology, Software, Visualization, Writing – review & editing. **Floriana De Angelis:** Data curation, Investigation, Validation, Writing – review & editing. **Ferran Prados:** Data curation, Investigation, Validation, Writing – review & editing. **Baris Kanber:** Data curation, Investigation, Validation, Writing – review & editing. **Alberto Calvi:** Data curation, Investigation, Validation, Writing – review & editing. **Arman Eshaghi:** Data curation, Investigation, Validation, Writing – review & editing. **Thalis Charalambous:** Data curation, Investigation, Validation, Writing – review & editing. **Rosa Cortese:** Data curation, Investigation, Validation, Writing – review & editing. **Declan T. Chard:** Conceptualization, Data curation, Funding acquisition, Investigation, Project administration, Supervision, Validation, Writing – review & editing. **Jeremy Chataway:** Conceptualization, Data curation, Funding acquisition, Investigation, Project administration, Supervision, Validation, Writing – review & editing. **Alan J. Thompson:** Conceptualization, Data curation, Funding acquisition, Investigation, Project administration, Supervision, Validation, Writing – review & editing. **Olga Ciccarelli:** Conceptualization, Data curation, Funding acquisition, Investigation, Project administration, Supervision, Validation, Writing – review & editing. **Claudia A.M. Gandini Wheeler-Kingshott:** Conceptualization, Data curation, Funding acquisition, Investigation, Project administration, Supervision, Validation, Writing – review & editing.

## Declaration of Competing Interest

The authors declare that they have no known competing financial interests or personal relationships that could have appeared to influence the work reported in this paper.
