# Supplementary material for: Spatial patterns of brain lesions assessed through covariance estimations of lesional voxels in multiple Sclerosis: The SPACE-MS technique
Source: Neuroimage Clin. 2021 Dec 2;33:102904. doi: 10.1016/j.nicl.2021.102904 (PMC8654632; doi:10.1016/j.nicl.2021.102904)
Supplement: Supplementary data 1 [file mmc1.docx]

**SUPPLEMENTARY MATERIAL**

**Supplementary figure 1**

**
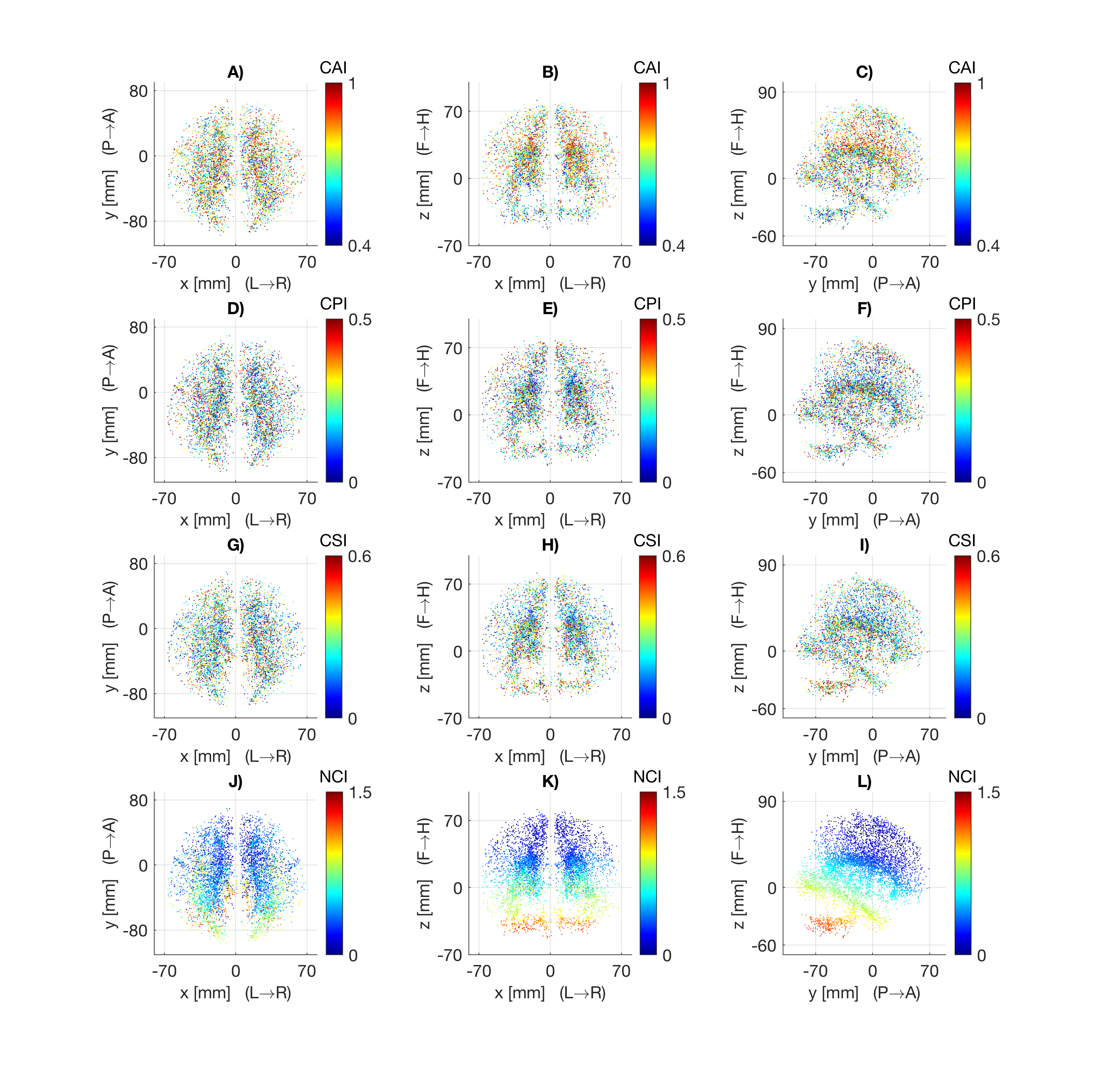
**

**(new) Supplementary Fig1. Variation of lesion-wise SPACE-MS metrics across the brain.** As an exploratory analysis, we computed the SPACE-MS metrics on individual lesions. This figure shows the spatial position of all lesions from baseline scans of the “Observational cohort” in MNI space, and colour-codes each lesion depending on the value of (lesion-wise) SPACE-MS metrics. As expected, lesion-wise NCI increases as the individual lesion becomes more caudal. In relation to CAI, CPI, and CSI, there is a trend towards lesions being more anisotropic (less isotropic) in the periventricular region. Future studies focusing on the analysis of lesion-wise metrics are warranted. *Abbreviations:* CAI: covariance anisotropy index; CPI: covariance planarity index; CSI: covariance sphericity index; NCI: neuraxis caudality index.
