# Supplementary material for: Spatial patterns of brain lesions assessed through covariance estimations of lesional voxels in multiple Sclerosis: The SPACE-MS technique
Source: Neuroimage Clin. 2021 Dec 2;33:102904. doi: 10.1016/j.nicl.2021.102904 (PMC8654632; doi:10.1016/j.nicl.2021.102904)
Supplement: Supplementary data 2 [file mmc2.docx]

**SUPPLEMENTARY MATERIAL**

**Supplementary tables**

**(new) Supplementary Table 1. Correlations between clinical scores measured at baseline and at follow-up**

| **Clinical measure** | **Correlation between BL and FU** | **p-value** |
| --- | --- | --- |
| EDSS | 0.8634 | <0.0001 |
| Inverse of TWT | 0.6826 | <0.0001 |
| Inverse of 9HPT | 0.8546 | <0.0001 |
| PASAT score | 0.7674 | <0.0001 |
| SDMT score | 0.8522 | <0.0001 |

**(new) Supplementary Table 1 (footnote).** *Abbreviations (in alphabetical order):* 9HPT: 9-hole peg test; BL: baseline; EDSS: expanded disability status scale; FU: follow-up; PASAT: paced auditory serial addition test; SDMT: symbol digit modalities test; TWT: 25-foot timed walk test.
