# Supplementary material for: Spatial patterns of brain lesions assessed through covariance estimations of lesional voxels in multiple Sclerosis: The SPACE-MS technique
Source: Neuroimage Clin. 2021 Dec 2;33:102904. doi: 10.1016/j.nicl.2021.102904 (PMC8654632; doi:10.1016/j.nicl.2021.102904)
Supplement: Supplementary data 3 [file mmc3.docx]

**SUPPLEMENTARY MATERIAL**

**Supplementary tables**

**(new) Supplementary Table 2. Spatial distribution metrics and clinical associations at baseline, using a backward elimination strategy for variable selection**

| **SPACE-MS metric^b^** | **Clinical variable^a^ (dependent variable)** | | | | |
| --- | --- | --- | --- | --- | --- |
|  | **EDSS score** | **Inverse of TWT** | **Inverse of 9HPT** | **PASAT score** | **SDMT score** |
| **NCI**  RC (95%CI), p-value  R^2^  % improvement | -0.163 (-1.175 to 0.848), p=0.751  R^2^=0.1129  0.1626% | 0.034 (-0.021 to 0.089), p=0.231  R^2^=0.2069  1.2502% | -0.016 (-0.028 to -0.004), **p=0.009**    R^2^=0.2450  11.9350% | 17.684 (0.081 to 35.287), **p=0.049**  R^2^=0.2302  2.9363% | -7.090 (-20.918 to 6.737), p=0.314  R^2^=0.3700  0.4780% |
| **Maximum lesion NCI**  RC (95%CI), p-value  R^2^  % improvement | 0.622 (0.078 to 1.165), **p=0.025**  R^2^=0.1218  8.0906% | -0.033 (-0.064 to -0.003), **p=0.032**  R^2^=0.2125  4.0033% | -0.009 (-0.015 to -0.002), **p=0.012**  R^2^=0.2298  4.9638% | -1.817 (-11.632 to 7.998), p=0.716  R^2^=0.2238  0.1005% | -11.695 (-19.568 to -3.821), **p=0.004**  R^2^=0.3827  3.9299% |
| **MCI**  RC (95%CI), p-value  R^2^  % improvement | 0.0005 (-0.0002 to 0.0013), p=0.174  R^2^=0.1160  2.9788% | -0.0003 (-0.0006 to -0.0001), **p=0.010**  R^2^=0.2055  5.8364% | -0.00001  (-0.00002 to -0.000004), **=0.004**  R^2^=0.2333  6.5761% | -0.010 (-0.024 to 0.003), p=0.133  R^2^=0.2274  1.7150% | -0.008 (-0.019 to 0.003), p=0.165  R^2^=0.3715  0.9088% |
| **CAI**  RC (95%CI), p-value  R^2^  % improvement | -0.797 (-1.499 to -0.095), **p=0.026**  R^2^=0.1217  7.9705% | 0.051 (0.012 to 0.089), **p=0.010**  R^2^=0.2162  5.7770% | 0.012 (0.003 to 0.020), **p=0.006**  R^2^=0.2316  5.7950% | 8.765 (-3.807 to 21.338), p=0.171  R^2^=0.2268  1.4204% | 12.881 (3.072 to 22.690), **p=0.010**  R^2^= 0.3796  3.0870% |
| **CPI**  RC (95%CI), p-value  R^2^  % improvement | -0.489 (-1.058 to 0.080), p=0.092  R^2^=0.1179  4.5888% | 0.024 (-0.007 to 0.056), p=0.124  R^2^=0.2086  2.0588% | 0.008 (0.001 to 0.015), **p=0.027**  R^2^=0.2273  3.8291% | 1.839 (-8.263557 11.94163), =0.721  R^2^=0.2238  0.0972% | 8.495 (0.422 to 16.568), **p=0.039**  R^2^=0.3755  1.9950% |
| **CSI**  RC (95%CI), p-value  R^2^  % improvement | 0.947 (0.333 to 1.560),  **p=0.003**  R^2^=0.1291  14.5824% | -0.054 (-0.088 to -0.020),  **p=0.002**  R^2^=0.2217  8.5130% | -0.013 (-0.020 to -0.006),  **p<0.001**  R^2^= 0.2396  9.4341% | -8.145 (-19.184 to 2.895),  p=0.148  R^2^=0.2272  1.5899% | -14.227 (-22.746 to -5.709),  **p=0.001**  R^2^=0.3864  4.9386% |
| **Model covariates**  (R^2^ without including the spatial distribution metric) | Lesion volume  Total intracranial volume  Age  Gender  Disease duration  Study centre  R^2^ =0.1127 | Lesion volume  Total intracranial volume  Age  Gender  Disease duration  Study centre  R^2^=0.2043 | Lesion volume  White matter volume  Age  Gender  Disease duration  Study centre  R^2^=0.2189 | Lesion volume  Total intracranial volume  Age  Study centre  R^2^=0.2236 | Lesion volume  White matter volume  Total intracranial volume  Gender  R^2^=0.3682 |

**(new) Supplementary Table 2. Footnote. a:** the EDSS score is measured in EDSS score units; the inverse of TWT and the inverse of 9HPT, in 1/s; and the PASAT and SDMT scores, in number of correct answers; **b:** all spatial distribution metrics are measured in dimensionless units except for MCI, which is measured in mm^2^; **c:** for MCI, the model included not only MCI but also MCI squared, which was significant: RC (95%CI): 2.46*10^-7^ (5.06*10^-8^ to 4.40*10^-7^), p=0.014; *Abbreviations (in alphabetical order):* % improvement: % improvement in model performance based on the R^2^; 9HPT: nine-hole peg test; CAI: covariance anisotropy index; CI: Confidence Interval; CPI: covariance planarity index; Max: maximum; NCI: neuraxis caudality index; PASAT: paced auditory serial addition test; R^2^: R-squared; RC: regression coefficient; SDMT: symbol digit modalities test; CSI: covariance sphericity index; MCI: mean covariance index; TWT: 25-foot timed walk test.
