# Supplementary material for: Spatial patterns of brain lesions assessed through covariance estimations of lesional voxels in multiple Sclerosis: The SPACE-MS technique
Source: Neuroimage Clin. 2021 Dec 2;33:102904. doi: 10.1016/j.nicl.2021.102904 (PMC8654632; doi:10.1016/j.nicl.2021.102904)
Supplement: Supplementary data 4 [file mmc4.docx]

**SUPPLEMENTARY MATERIAL**

**Supplementary tables**

**new) Supplementary Table 3. Prediction of future disability using spatial distribution metrics, using a backward elimination strategy for variable selection**

| **SPACE-MS metric^b^** | **Clinical variable^a^ at follow-up (dependent variable)** | | | | |
| --- | --- | --- | --- | --- | --- |
|  | **EDSS score** | **Inverse of TWT** | **Inverse of 9HPT** | **PASAT score** | **SDMT score** |
| **NCI**  RC (95%CI), p-value  R^2^  % improvement | 1.207 (0.322 to 2.092), **p=0.008**  R^2^=0.6200  0.7330% | -0.053 (-0.102 to -0.004), **p=0.033**  R^2^=0.5567  0.8967% | -0.0003 (-0.008 to 0.008), =0.949  R^2^=0.7492  0.0003% | 3.965 (-9.283 to 17.213), p=0.557  R^2^=0.6598  0.0415% | -4.372 (-13.621 to 4.876), =0.353  R^2^=0.8062  0.0627% |
| **Maximum lesion NCI**  RC (95%CI),  p-value  R^2^  % improvement | 0.516 (0.038 to 0.995),  **p=0.035**  R^2^=0.6171  0.2767% | -0.023 (-0.050 to 0.005),  p=0.109  R^2^=0.5545  0.5063% | -0.004 (-0.008 to 0.001),  p=0.094  R^2^=0.7508  0.2135% | 1.377 (-5.969 to 8.722),  p=0.713  R^2^=0.6596  0.0163% | -8.448 (-13.761 to -3.136),  **p=0.002**  R^2^=0.8113  0.6904% |
| **MCI**  RC (95%CI), p-value  R^2^  % improvement | -0.0002 (-0.0008 to 0.0004),  p=0.422  R^2^=0.6124  -0.4926% | 9.81*10^-6^  (-0.00002 to 0.00004),  p=0.555  R^2^=0.5521  0.0688% | -2.26 *10^-7^  (-5.61 *10^-6^ to 5.16 *10^-6^),  p=0.934  R^2^=0.7492  0.0005% | -0.002 (-0.012 to 0.008),  p=0.685  R^2^=0.6597  0.0198% | -0.002 (-0.008 to 0.005),  p=0.590  R^2^=0.8059  0.0211% |
| **CAI**  RC (95%CI), p-value  R^2^  % improvement | -0.544 (-1.162 to 0.074),  p=0.084  R^2^=0.6157  0.0488% | 0.016 (-0.019 to 0.051),  p=0.380  R^2^=0.5526  0.1525% | 0.007 (0.002 to 0.013),  **p=0.013**  R^2^=0.7527  0.4666% | -1.151 (-10.587 to 8.285),  p=0.811  R^2^=0.6596  0.0069% | 8.475 (1.852 to 15.098),  **p=0.012**  R^2^=0.8093  0.4516% |
| **CPI**  RC (95%CI), p-value  R^2^  % improvement | -0.117 (-0.622 to 0.388),  p=0.649  R^2^=0.6137  -0.2867% | 0.019 (-0.010 to 0.047),  p=0.202  R^2^=0.5535  0.3208% | 0.003 (-0.002 to 0.007),  p=0.282  R^2^=0.7498  0.0883% | -3.664 (-11.214 to 3.887),  p=0.341  R^2^=0.6602  0.1090% | 2.835 (-2.805 to 8.475),  p=0.323  R^2^=0.8063  0.0708% |
| **CSI**  RC (95%CI),  p-value  R^2^  % improvement | 0.495 (-.0570642 1.046575),  p=0.079  R^2^=0.6169  0.2398% | -0.025 (-0.057 to 0.006),  p=0.113  R^2^=0.5545  0.4955% | -0.008 (-0.013 to -0.003),  **p=0.003**  R^2^=0.7543  0.6863% | 3.892 (-4.342 to 12.126),  p=0.353  R^2^=0.6602  0.1034% | -8.590 (-14.594 to -2.585),  **p=0.005**  R^2^=0.8102  0.5618% |
| **Model covariates**  (Model R^2^ without including the spatial distribution metric) | Clinical variable at baseline  Lesion volume  Age  Gender  Study centre  R^2^=0.6154 | Clinical variable at baseline  Lesion volume  Age  Study centre  R^2^=0.5517 | Clinical variable at baseline  Lesion volume  Age  Study centre  R^2^=0.7492 | Clinical variable at baseline  Lesion volume  White matter volume  Total intracranial volume  Age  Study centre  R^2^=0.6595 | Clinical variable at baseline  Lesion volume  Grey matter volume  Age  Study centre  R^2^=0.8057 |

**(new) Supplementary Table 3. Footnote. a:** the EDSS score is measured in EDSS score units; the inverse of TWT and the inverse of 9HPT, in 1/s; and the PASAT and SDMT scores, in number of correct answers; **b:** all spatial distribution metrics are measured in dimensionless units except for MCI, which is measured in mm^2^; *Abbreviations (in alphabetical order):* % improvement: % improvement in model performance based on the R^2^; 9HPT: nine-hole peg test; CAI: covariance anisotropy index; CI: Confidence Interval; CPI: covariance planarity index; NCI: neuraxis caudality index; PASAT: paced auditory serial addition test; R^2^: R-squared; RC: regression coefficient; SDMT: symbol digit modalities test; CSI: covariance sphericity index; MCI: mean covariance index; TWT: 25-foot timed walk test.
