# Supplementary material for: Spatial patterns of brain lesions assessed through covariance estimations of lesional voxels in multiple Sclerosis: The SPACE-MS technique
Source: Neuroimage Clin. 2021 Dec 2;33:102904. doi: 10.1016/j.nicl.2021.102904 (PMC8654632; doi:10.1016/j.nicl.2021.102904)
Supplement: Supplementary data 5 [file mmc5.docx]

**SUPPLEMENTARY MATERIAL**

**Supplementary tables**

**(new) Supplementary Table 4. Longitudinal models with a reparameterisation of the ‘time’ variable**

| **SPACE-MS metric** (in dimensionless units, unless otherwise specified) | ***Original LME model***  **‘Time’ variable:**  **within-study follow-up time (in years), adjusting for age and disease duration**  *RC (95%CI), p-value* | ***New LME model 1***  **‘Time’ variable:**  **disease duration (in years), adjusting for age**  *RC (95%CI), p-value* | ***New LME model 2a***  **‘Time’ variable:**  **age (in years), adjusting for disease duration**  *RC (95%CI), p-value* | ***New LME model 2b***  **‘Time’ variable:**  **age (in years), with no adjustment for disease duration**  *RC (95%CI), p-value* |
| --- | --- | --- | --- | --- |
| NCI | -0.0007  (-0.0013 to -0.0002),  **p=0.012** | -0.0008  (-0.0013 to -0.0003),  **p=0.001**  **Quadratic model:**  RC [disease duration] = -0.0019 (-0.0032 to -0.0006), p=0.004  RC [disease duration^2^] = 0.00002 (-2.05•10^-06^ to 0.00005), p=0.073 | -0.0007  (-0.0012 to -0.0002),  **p=0.010**  **Quadratic model:**  RC [age] = -0.0049 (-0.0093 to -0.0006), p=0.026    RC [age^2^]_= 0.00004 (-4.79•10^-07^ to 0.00008), p=0.053 | -0.0007  (-0.0012 to -0.0002),  **p=0.004**  **Quadratic model:**  RC [age] = -0.0047 (-0.0090 to -0.0004), p=0.033    RC [age^2^]_= 0.00004 (-3.01•10^-06^ to 0.00007), p=0.071 |
| Maximum lesion NCI | 0.0011  (0.0002 to 0.0021),  **p= 0.018** | 0.0008  (0.0001 to 0.0016),  **p=0.037** | 0.0008  (-0.0001 to 0.0017),  p=0.0711 | 0.0008  (-0.0001 to 0.0016),  p=0.080 |
| MCI (in mm^2^) | 0.2854  (-0.5238 to 1.0946),  p=0.489 | 0.2425  (-0.5005 to 0.9855),  p=0.522 | 0.4708  (-0.3519 to 1.2936),  p=0.2620 | 0.4430  (-0.3564 to 1.2424),  p=0.277 |
| CAI | -0.0008  (-0.0013 to -0.0002),  **p=0.005** | -0.0007  (-0.0013 to -0.0001),  **p=0.014** | -0.0005  (-0.0011 to 0.0002),  p=0.1539  **Quadratic model:**  RC [age] = -0.0069 (-0.0122 to -0.0016), p=0.011  RC [age^2^]_= 0.00006 (0.00001 to 0.0001), p=0.016 | -0.0004  (-0.0011 to 0.0002),  p=0.158  **Quadratic model:**  RC [age] = -0.0069 (-0.0122 to -0.0016), p=0.010  RC [age^2^]_= 0.00006 (0.00001 to 0.00010), p=0.015 |
| CPI | 0.0007  (-0.0003 to 0.0016),  p=0.162 | 0.0004  (-0.0005 to 0.0013),  p=0.422 | 0.0029  (0.0008 to 0.0050),  **p=0.006** | 0.0031  (0.0011 to 0.0052),  **p=0.003** |
| CSI | 0.0005  (-0.0002 to 0.0011),  p=0.191 | 0.0005  (-0.0002 to 0.0012),  p=0.181 | 0.0015  (0.0004 to 0.0025),  **p=0.005** | 0.0016  (0.0006 to 0.0026),  **p=0.002** |
| Covariates (apart from the ‘time’ variable) | Lesion volume at BL  Changes in lesion volume from study BL to follow-up  GM volume  WM volume  Scanning centre  Sex  Age at BL  Disease duration at BL | Lesion volume at BL  Changes in lesion volume from study BL to follow-up  GM volume  WM volume  Scanning centre  Sex  Age at BL | Lesion volume at BL  Changes in lesion volume from study BL to follow-up  GM volume  WM volume  Scanning centre  Sex  Disease duration at BL | Lesion volume at BL  Changes in lesion volume from study BL to follow-up  GM volume  WM volume  Scanning centre  Sex |

**(new) Supplementary Table 4 (footnote).** Longitudinal mixed-effects (LME) models reflecting the evolution of each one of the SPACE-MS metrics over time, using different time variables. *Abbreviations (in alphabetical order):* BL: baseline; CAI: covariance anisotropy index; CI: Confidence Interval; CPI: covariance planarity index; CSI: covariance sphericity index; MCI: mean covariance index. NCI: neuraxis caudality index; RC: regression coefficient;
