# Supplementary material for: Spatial patterns of brain lesions assessed through covariance estimations of lesional voxels in multiple Sclerosis: The SPACE-MS technique
Source: Neuroimage Clin. 2021 Dec 2;33:102904. doi: 10.1016/j.nicl.2021.102904 (PMC8654632; doi:10.1016/j.nicl.2021.102904)
Supplement: Supplementary data 6 [file mmc6.docx]

**SUPPLEMENTARY MATERIAL**

**Supplementary tables**

**(new) Supplementary Table 5. Association between whole-brain and lesion-wise (*mean across lesions*) SPACE-MS metrics at baseline: partial correlation coefficients**

| ***Mean* lesion-wise SPACE-MS metrics ↓** | **Mean (SD)** | **Whole-brain lesion-mask SPACE-MS metrics^#^** | | | | |
| --- | --- | --- | --- | --- | --- | --- |
|  |  | **NCI** | **MCI** | **CAI** | **CPI** | **CSI** |
| **NCI*** | 0.4848 (0.0805) | 0.4602 p<0.001 | -0.0318 p=0.478 | -0.0308 p=0.492 | -0.2289 p<0.001 | 0.1552 p<0.001 |
| **MCI** | 7.3897 (5.6374) | -0.1799  p<0.001 | -0.0134  p=0.765 | -0.1394  p=0.002 | 0.2417  p<0.001 | -0.0064  p=0.887 |
| **CAI** | 0.7219 (0.0487) | -0.0545  p=0.224 | -0.0289  p=0.520 | 0.1600  p<0.001 | -0.0495  p=0.2696 | -0.1204  p=0.007 |
| **CPI** | 0.2877 (0.0316) | -0.0519  p=0.247 | 0.1191  p=0.008 | 0.0052  p=0.909 | 0.0590  p=0.1881 | -0.0436  p=0.330 |
| **CSI** | 0.2597  (0.0606) | 0.0760  p=0.090 | -0.0220  p=0.624 | -0.1572  p<0.001 | 0.0198  p=0.6581 | 0.1349  p=0.003 |
| **Individual lesion volume^£^** | 280.8457 (341.973) | -0.1964  p<0.001 | -0.0370  p=0.409 | -0.1535  p<0.001 | 0.2454  p<0.001 | 0.0001  p=0.998 |

**(new) Supplementary Table 5. Footnote.** # partial correlation coefficient, after adjusting for scanning centre; *please notice that this is not the maximum lesion NCI, which is the metric already presented in the paper and which represents the caudality of the most caudal brain lesion; £ this metric is not a spatial distributional metric, but a volumetric measure, which has been included for reference. *Abbreviations:* CAI: covariance anisotropy index; CPI: covariance planarity index; CSI: covariance sphericity index; NCI: neuraxis caudality index.
