# Supplementary material for: Spatial patterns of brain lesions assessed through covariance estimations of lesional voxels in multiple Sclerosis: The SPACE-MS technique
Source: Neuroimage Clin. 2021 Dec 2;33:102904. doi: 10.1016/j.nicl.2021.102904 (PMC8654632; doi:10.1016/j.nicl.2021.102904)
Supplement: Supplementary data 7 [file mmc7.docx]

**SUPPLEMENTARY MATERIAL**

**Supplementary tables**

**(new) Supplementary Table 6. Ability of *native-space* and *MNI-space* SPACE metrics to predict concurrent disability in a subset of patients (‘Observational cohort’)**

|  | **Image space** | **Clinical variable at baseline^#^ (dependent variable)** | |
| --- | --- | --- | --- |
|  |  | **EDSS score** | **SDMT score** |
| **NCI**  RC (95%CI), p-value  *Model R^2^ (%I)* | *Native (3DT1) space* | -5.1775 (-14.7296 to 4.3745), p=0.274  R^2^=0.1762 | -24.6859 (-98.0643 to 48.6925), p=0.493  R^2^=0.3581 |
|  | *MNI space* | -6.4523 (-13.9154 to 1.0108), p=0.087  R^2^=0.2293 | 3.8245 (-53.8967 to 61.5458), p=0.892  R^2^=0.3296 |
| **Maximum lesion NCI**  RC (95%CI), p-value  *Model R^2^ (%I)* | *Native (3DT1) space* | **5.6392 (2.1607 to 9.1177), p=0.003**  R^2^**=0.4090** | **-**6.5875 (-34.4092 to 21.2342), p=0.628  R^2^=0.35104775 |
|  | *MNI space* | **5.7877 (1.5573 to 10.0180), p=0.009**  R^2^**=0.3448** | -10.9094 (-43.2998 to 21.4811), p=0.492  R^2^=0.3436 |
| **MCI**  RC (95%CI), p-value  *Model R^2^ (%I)* | *Native (3DT1) space* | -0.0029 (-0.0109 to 0.0051), p=0.459  R^2^=0.1532 | -0.0039 (-0.0573 to 0.0495), p=0.881  R^2^=0.3446 |
|  | *MNI space* | -0.0019 (-0.0066 to 0.0028), p=0.417  R^2^=0.1512 | 0.0004 (-0.0321 to 0.0329), p=0.978  R^2^=0.3291 |
| **CAI**  RC (95%CI), p-value  *Model R^2^ (%I)* | *Native (3DT1) space* | **-6.7565 (-11.2865 to -2.2264), p=0.005**  R^2^**=0.3786** | 22.4915 (-12.4902 to 57.4732), p=0.196  R^2^=0.3930 |
|  | *MNI space* | **-6.4762 (-10.0839 to -2.8686), p=0.001**  R^2^**=0.4447** | 25.6900 (-2.9524 to 54.3325), p=0.076  R^2^=0.4202 |
| **CPI**  RC (95%CI), p-value  *Model R^2^ (%I)* | *Native (3DT1) space* | 1.879326 (-2.5625 to 6.3211), p=0.391  R^2^=0.1600 | 16.5518 (-12.5073 to 45.6109), p=0.250  R^2^=0.3831 |
|  | *MNI space* | 2.3489 (-1.2941 to 5.9919), p=0.196  R^2^=0.1871 | **26.4845 (3.2951 to 49.6739), p=0.027**  R^2^**=0.4654** |
| **CSI**  RC (95%CI), p-value  *Model R^2^* | *Native (3DT1) space* | **5.2993 (0.8903 to 9.7084), p=0.021**  R^2^**=0.3102** | -28.5271 (-59.4185 to 2.3644), p=0.069  R^2^=0.4377 |
|  | *MNI space* | **4.2431 (0.5106 to 7.9755), p=0.028**  R^2^**=0.2899** | **-33.6254 (-57.5302 to -9.7207), p=0.008**  R^2^**=0.5162** |

**(new) Supplementary Table 6. Footnote. a:** the EDSS score is measured in EDSS score units; the inverse of TWT and the inverse of 9HPT, in 1/s; and the PASAT and SDMT scores, in number of correct answers; **b:** all spatial distribution metrics are measured in dimensionless units except for MCI, which is measured in mm^2^; *Abbreviations (in alphabetical order):* 9HPT: nine-hole peg test; Adj R^2^: adjusted R-squared; CAI: covariance anisotropy index; CI: Confidence Interval; CPI: covariance planarity index; NCI: neuraxis caudality index; PASAT: paced auditory serial addition test; RC: regression coefficient; SDMT: symbol digit modalities test; CSI: covariance sphericity index; MCI: mean covariance index; TWT: 25-foot timed walk test.
