# Supplementary material for: Spatial patterns of brain lesions assessed through covariance estimations of lesional voxels in multiple Sclerosis: The SPACE-MS technique
Source: Neuroimage Clin. 2021 Dec 2;33:102904. doi: 10.1016/j.nicl.2021.102904 (PMC8654632; doi:10.1016/j.nicl.2021.102904)
Supplement: Supplementary data 8 [file mmc8.docx]

**SUPPLEMENTARY MATERIAL**

**Supplementary tables**

**(new) Supplementary Table 7. Ability of *native-space* and *MNI-space* SPACE metrics to predict future disability in a subset of patients (‘Observational cohort’)**

|  | **Image space** | **Clinical variable at follow-up^#^ (dependent variable)** | |
| --- | --- | --- | --- |
|  |  | **EDSS score** | **SDMT score** |
| **NCI**  RC (95%CI), p-value  *Model R^2^ (%I)* | *Native (3DT1) space* | 0.3470 (-2.0746 to 2.7686), p=0.771  R^2^=0.9100 | -7.8972 (-55.6039 to 39.8095), p=0.723  R^2^=0.7461 |
|  | *MNI space* | -0.2665 (-2.323 to 1.7900), p=0.792  R^2^=0.9099 | 11.2964 (-25.9430 to 48.5358), p=0.518  R^2^=0.7359 |
| **Maximum lesion NCI**  RC (95%CI), p-value  *Model R^2^ (%I)* | *Native (3DT1) space* | 0.7815 (-0.2500 to 1.8130), p=0.132  R^2^=0.9171 | -0.0028 (-0.0328 to 0.0272), p=0.840  R^2^=0.7840 |
|  | *MNI space* | 0.9248 (-0.1250 to 1.9745), p=**0.082**  R^2^=0.9194 | -12.1223 (-30.4792 to 6.2345), p=0.174  R^2^=0.7694 |
| **MCI**  RC (95%CI), p-value  *Model R^2^ (%I)* | *Native (3DT1) space* | 0.0002 (-0.0017 to 0.0022), p=0.800  R^2^=0.9099 | 0.0137 (-0.0521 to 0.0794), p=0.630  R^2^=0.7441 |
|  | *MNI space* | -0.0005 (-0.0017 to 0.0008), p=0.436  R^2^=0.9117 | -0.0014 (-0.0309 to 0.0281), p=0.919  R^2^=0.7254 |
| **CAI**  RC (95%CI), p-value  *Model R^2^ (%I)* | *Native (3DT1) space* | -0.2832 (-1.7423 to 1.1759), p=0.694  R^2^=0.9102 | 16.9948 (-5.5700 to 39.5597), p=0.126  R^2^=0.7944 |
|  | *MNI space* | -0.2341 (-1.5002 to 1.0319), p=0.707  R^2^=0.9101 | 18.4383 (-0.1029 to 36.9794), p=**0.051**  R^2^=0.8085 |
| **CPI**  RC (95%CI), p-value  *Model R^2^ (%I)* | *Native (3DT1) space* | -0.0464 (-1.2063 to 1.1135), p=0.935  R^2^=0.9097 | 2.7817 (-18.8431 to 24.4064), p=0.782  R^2^=0.7449 |
|  | *MNI space* | -0.1515 (-1.1606 to 0.8576), p=0.760  R^2^=0.9100 | 8.4221 (-10.8937 to 27.7380), p=0.358  R^2^=0.7464 |
| **CSI**  RC (95%CI), p-value  *Model R^2^ (%I)* | *Native (3DT1) space* | 0.3481 (-0.9630 to 1.6592), p=0.590  R^2^=0.9107 | -18.2982 (-37.1052 to 0.5089), p=**0.055**  R2=0.8187 |
|  | *MNI space* | 0.2726 (-0.7852 to 1.3305), p=0.601  R^2^=0.9106 | **-19.7636 (-33.7513 to -5.7759), p=0.010**  R^2^**=0.8537** |

**(new) Supplementary Table 7. Footnote. a:** the EDSS score is measured in EDSS score units; the inverse of TWT and the inverse of 9HPT, in 1/s; and the PASAT and SDMT scores, in number of correct answers; **b:** all spatial distribution metrics are measured in dimensionless units except for MCI, which is measured in mm^2^; *Abbreviations (in alphabetical order):* 9HPT: nine-hole peg test; Adj R^2^: adjusted R-squared; CAI: covariance anisotropy index; CI: Confidence Interval; CPI: covariance planarity index; NCI: neuraxis caudality index; PASAT: paced auditory serial addition test; RC: regression coefficient; SDMT: symbol digit modalities test; CSI: covariance sphericity index; MCI: mean covariance index; TWT: 25-foot timed walk test.
